# Gut Commensal *Barnesiella Intestinihominis* Ameliorates Hyperglycemia and Liver Metabolic Disorders

**DOI:** 10.1002/advs.202411181

**Published:** 2024-12-31

**Authors:** Ye Zhang, Dong Xu, Xuyi Cai, Xue Xing, Xin Shao, Ailing Yin, Yanyan Zhao, Mengyuan Wang, Yu‐nuo Fan, Boao Liu, Hua Yang, Wei Zhou, Ping Li

**Affiliations:** ^1^ State Key Laboratory of Natural Medicines China Pharmaceutical University Nanjing 211198 China; ^2^ Nanjing Hospital of Chinese Medicine affiliated to Nanjing University of Chinese Medicine Nanjing 210022 China; ^3^ Department of Endocrinology The First Affiliated Hospital of Zhengzhou University Zhengzhou 450052 China

**Keywords:** acetate, fibroblast growth factor 21, gut microbiota, histone deacetylase 9, puerarin, type 2 diabetes

## Abstract

Recent studies have highlighted the role of the gut microbiota in type 2 diabetes (T2D). Improving gut microbiota dysbiosis can be a potential strategy for the prevention and management of T2D. Here, this work finds that the abundance of *Barnesiella intestinihominis* is significantly decreased in the fecal of T2D patients from 2‐independent centers. Oral treatment of live *B. intestinihominis* (LBI) considerably ameliorates hyperglycemia and liver metabolic disorders in HFD/STZ‐induced T2D models and db/db mice. LBI‐derived acetate has similar protective effects against T2D. Mechanistically, acetate enhances fibroblast growth factor 21 (FGF21) through inhibition of histone deacetylase 9 (HDAC9) to increase H3K27 acetylation at the FGF21 promoter. The screening puerarin from Gegen Qinlian decoction in a gut microbiota‐dependent manner improved hyperglycemia and liver metabolic disorders by promoting the growth of *B. intestinihominis*. This study suggests that gut commensal *B. intestinihominis* and puerarin, respectively have the potential as a probiotic and prebiotic in the treatment of T2D.

## Graphical abstract

This study finds that the abundance of *B. intestinihominis* is decreased in the fecal of T2D mice and patients from 2‐independent centers. Oral administration of live *B. intestinihominis* attenuates hyperglycemia and liver metabolic disorders in T2D mice. *B. intestinihomini‐*derived acetate ameliorates T2D by increasing FGF21 through inhibition of HDAC9 to enhance H3K27 acetylation at the FGF21 promoter. Puerarin is a promising prebiotic agent for T2D treatment by promoting the growth of *B. intestinihominis*.

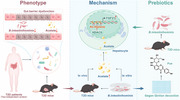

## 1 Introduction

Type 2 diabetes (T2D) is a chronic disease, characterized by hyperglycemia arising from insulin resistance and relative insulin deficiency that affects more than 400 million people worldwide.^[^ ^1^ ^]^ As the global prevalence of T2D has increased dramatically, there is an urgent need for enhanced prevention and management strategies.^[^ ^2^ ^]^ The liver plays a key role in the maintenance of glucose homeostasis.^[^ ^3^, ^4^ ^]^ Liver metabolic disorders are risk factors for the development of T2D.^[^ ^5^ ^]^ Under T2D conditions, liver dysfunction can lead to increased hepatic gluconeogenesis, inhibited glycogen storage, increased free fatty acids, and steatolysis, resulting in lipotoxicity damage.^[^ ^6^, ^7^ ^]^ Furthermore, hepatocellular injury and fatty changes in the liver aggravate glucose homeostasis.^[^ ^8^ ^]^ These mechanistic findings are consistent with meta‐analyses showing that metabolic dysfunction‐associated fatty liver disease (MAFLD) is a predictor of the development of peripheral insulin resistance and T2D.^[^ ^9^, ^10^ ^]^ Therefore, the improvement of liver metabolic disorders to combat T2D has become highly important.

Accumulating evidence has shown that the gut microbiota played a crucial role in the progression of T2D.^[^ ^11^ ^]^ Manipulation of the gut microbiota may provide a new and safe therapeutic modality for combating T2D. The dipeptidyl peptidase 4 from *Bacteroides thetaiotaomicron* degrades the glucagon‐like peptide‐1 (GLP‐1) to affect blood glucose homeostasis.^[^ ^12^ ^]^ In contrast, supplementation with *Clostridium sp* could increase ursodeoxycholic acid levels to improve glucose intolerance by increasing GLP‐1 secretion.^[^ ^13^ ^]^ The gut‒liver axis communicates mainly through the portal vein, which promotes bacterial messengers to regulate liver glucose and lipid metabolism.^[^ ^14^, ^15^ ^]^ *Prevotella copri* and *Bacteroides vulgatus* have been identified as the main species that drive the biosynthesis of branched‐chain amino acids, a group of metabolites that contribute to liver metabolic disorders.^[^ ^16^, ^17^, ^18^ ^]^ An increased level of bacteria‐derived imidazole propionate deteriorates hepatic insulin resistance by activating rapamycin complex 1.^[^ ^19^ ^]^ The gut microbiota produces secondary bile acids that improve hepatic inflammation, glucose, and lipid homeostasis via the farnesoid X receptor and the transmembrane G‐coupled protein receptor 5.^[^ ^20^, ^21^, ^22^ ^]^ Thus, prevention of gut microbiota dysbiosis and restoration of homeostasis could be potential strategies for the prevention and management of T2D.

In our study, we found that the abundance of the gut commensal, *Barnesiella intestinihominis* (*B. intestinihominis*), in the fecal samples of T2D mice and patients from two‐independent centers was significantly decreased. *B. intestinihomini* is an anaerobic obligate gram‐negative commensal, and few reports on the role of *B. intestinihomini* in T2D exist.^[^ ^23^ ^]^ We demonstrated that oral administration of live *B. intestinihomini* significantly ameliorated hyperglycemia and liver metabolic disorders in HFD/STZ induced T2D model and db/db mice. Untargeted and targeted metabolomics, together with transcriptomics, revealed that *B. intestinihomini*‐derived acetate ameliorated T2D by increasing fibroblast growth factor 21 (FGF21) in hepatocytes. Acetate inhibited histone deacetylase 9 (HDAC9) to increase H3K27 acetylation at the FGF21 promoter. The screened natural product puerarin promoted the growth of *B. intestinihomini* to ameliorate hyperglycemia and liver metabolic disorders in a gut microbiota‐dependent manner. Thus, treatment of *B. intestinihominis* as a probiotic and puerarin as a prebiotic agent might be promising strategies for treating T2D.

## 2 Results

### 2.1 The Reduced Abundance of *B. Intestinihominis* in T2D Mice and Patients

**Figure 1:**
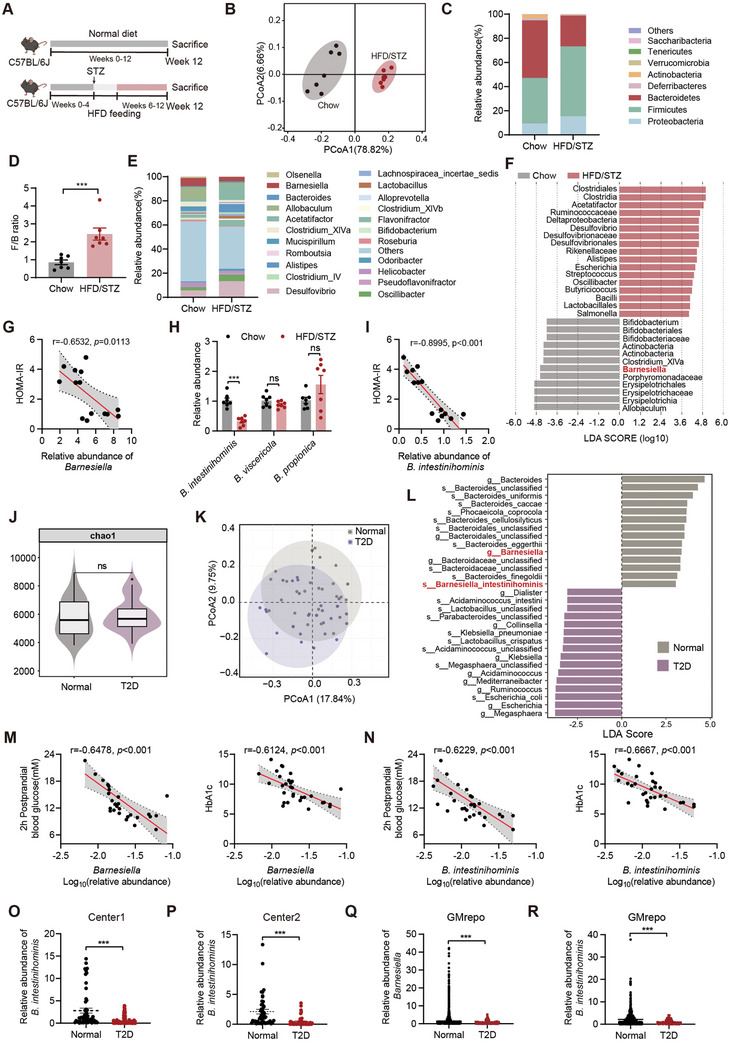
The reduced abundance of *B. intestinihominis* in T2D mice and patients. A) Schematic diagram of the study design. B) Weighted UniFrac PCoA analysis based on the OTU data of chow and HFD/STZ groups (*n* = 7). C) The abundance of bacteria at the phylum level (percentage of total bacteria). D) *Firmicutes*‐to‐*Bacteroidetes* ratio (*n* = 7). E) The abundance of bacteria at the genus level (percentage of total bacteria). F) LDA score represents the taxonomic data with significant differences between chow and HFD/STZ groups (*p *< 0.05, LDA scores > 4). G) Pearson's analysis of the correlations between the fecal *Barnesiella* abundance and HOMA‐IR index. H) The abundance of *B. intestinihominis*, *B. viscericola* and *B. propionica* was assessed by qPCR (*n* = 7). I) Pearson's analysis of the correlations between the fecal *B. intestinihominis* abundance and HOMA‐IR index. J) Chao index from shotgun metagenomics. K) PCoA analysis for the human fecal from T2D patients (*n* = 31) and healthy subjects (*n* = 30). L) LDA score represents the taxonomic data with significant differences between T2D patients (*n* = 31) and healthy subjects (*n* = 30) in genus and species (*p *< 0.05, LDA scores > 3). M) Pearson's analysis of the correlations between the fecal *Barnesiella* abundance from shotgun metagenomics and 2hPBG and HbA1c, respectively. N) Pearson's analysis of the correlations between the fecal *B. intestinihominis* abundance from shotgun metagenomics and 2hPBG and HbA1c, respectively. O) The abundance of *B. intestinihominis* using T2D patients (*n* = 150) and healthy subjects (*n* = 56) from microbiota validation set 1, assessed by qPCR. P) The abundance of *B. intestinihominis* using T2D patients (*n* = 57) and healthy subjects (*n* = 47) from microbiota validation set 2, assessed by qPCR. Q) The abundance of *Barnesiella* of T2D patients (*n* = 233) and healthy subjects (*n* = 8697) from GMrepo Database. R) The abundance of *B. intestinihominis* of T2D patients (*n* = 112) and healthy subjects (*n* = 3356) from GMrepo Database. Data were shown as mean ± SEM. Statistical analysis was performed by a two‐tailed Student's t‐test (D and H) or Mann‐Whitney U test (O‐R). ***p < 0.001; ns, no significance.

To identify the potential beneficial microbiome in T2D, a mouse model of HFD/STZ was utilized (**Figure** 1).^[^ ^24^ ^]^ As shown in Figure S1A,B (Supporting Information), body weight did not differ among the chow and HFD/STZ groups. HFD/STZ significantly affected glucose homeostasis, as demonstrated by increased fasting blood glucose levels, serum insulin levels, homeostasis model assessment of insulin resistance (HOMA‐IR) index, and impaired oral glucose tolerance test (OGTT) and insulin tolerance test (ITT) (Figure S1C–G, Supporting Information). In addition, T2D patients are usually accompanied by hypercholesterolemia and lipid accumulation in the liver. Increased liver weight, liver weight/body weight ratios, and serum aminotransferase (ALT), aspartate aminotransferase (AST) levels, total cholesterol (TC) and triglyceride (TG) levels indicated that HFD/STZ mice had hepatic steatosis and damage, as observed in H&E‐stained liver sections and a higher NAFLD activity score (NAS) (Figure S1H–N, Supporting Information). These results suggested that the HFD/STZ‐induced mouse model mimicked the clinical phenotype of T2D patients.

We next performed 16S rRNA gene sequencing analysis on fecal samples from chow mice and HFD/STZ mice. HFD/STZ did not affect α‐diversity (Figure S2A–C, Supporting Information), whereas principal coordinate analysis (PCoA) revealed a fecal microbial separation between the 2 groups (Figure 1B). HFD/STZ mice presented clear alterations at the phylum and genus levels and presented a higher *Firmicutes*‐to‐*Bacteroidetes* ratio (Figure 1C–E). To determine which gut microbiota members had a dominant role in T2D, we performed the linear discriminant analysis effect size (LEfSe). At the genus level, the abundances of *Allobaculum*, *Barnesiella*, *Clostridium_XlVa*, and *Bifidobacterium* were decreased, whereas the abundances of *Acetatifactor*, *Desulfovibrio*, and *Alistipes* were increased in HFD/STZ mice (Figure 1F). Importantly, we found that the abundance of the genus *Barnesiella* was negatively associated with multiple metabolic parameters (including fasting blood glucose, HOMA‐IR index, OGTT, ITT, TC, and TG levels) (Figure 1G; Figure S2D–I, Supporting Information). We identified 3 species (*B. intestinihominis*, *B.viscericola*, and *B.propionica*) among the genus *Barnesiella*. *B. intestinihominis* was significantly decreased in the fecal samples from HFD/STZ mice, and the abundance of *B. intestinihominis* was inversely associated with multiple metabolic parameters (Figure 1H,I; Figure S2J–N, Supporting Information).

Furthermore, we conducted shotgun metagenomics by 30 healthy subjects and 31 T2D patients from Nanjing Hospital of Chinese Medicine affiliated to Nanjing University of Chinese Medicine and found significant differences in the abundance of the gut microbiota. (Figure 1J,K; Figure S3A,B, Table S1, Supporting Information). LEfSe analysis revealed that the abundance of *Barnesiella* in the genus level and its species‐*B. intestinihominis* were decreased in the fecal samples from T2D patients, and a significantly negative correlation with the 2 h postprandial blood glucose (2hPBG) and Hemoglobin A1c (HbA1c) (Figure 1M,N; Figure S3C,D, Supporting Information). Additionally, our qPCR results demonstrated a significant decrease in *B. intestinihominis* abundance in the fecal samples from T2D patients (microbiota validation sets 1 and 2, Figure 1O,P; Figure S4 and Table S2, Supporting Information). Besides, we compared the fecal abundances of *Barnesiella* genus and *B. intestinihominis* in the GMrepo database.^[^ ^25^ ^]^ The T2D patients had lower levels of *Barnesiella* and *B. intestinihominis* (Figure 1Q,R) than the healthy subjects. Thus, *B. intestinihominis* may be related to the pathological progression of T2D.

### 2.2 Oral Administration of Live *B. Intestinihomini* Ameliorated Hyperglycemia and Liver Metabolic Disorders in HFD/STZ Mice

**Figure 2:**
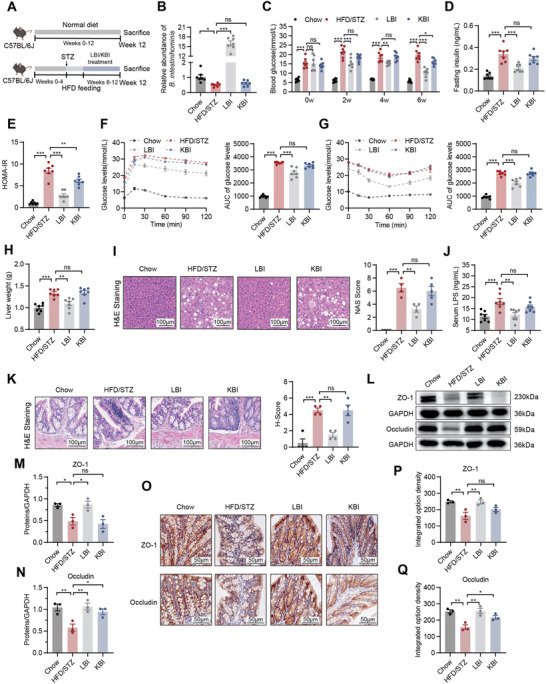
Oral administration of LBI attenuates metabolic disorder and gut barrier dysfunction in HFD/STZ mice. A) A schematic diagram showing the procedure of HFD/STZ mice treated with LBI and KBI. B) The abundance of *B. intestinihominis* assessed by qPCR (*n* = 7). C) Fasting blood glucose levels at 2, 4 and 6 weeks (*n* = 7). D) Insulin levels (*n* = 7). E) HOMA‐IR index (*n* = 7). F‐G) OGTT F) and ITT G) with AUC (*n* = 7). H) Liver weight (*n* = 7). I) Representative photomicrographs of liver H&E staining and histological scores (scale bar, 100 µm, *n* = 4–5). J) The serum LPS levels (*n* = 7). K) Representative photomicrographs of colon H&E staining and histological scores (scale bar, 100 µm, *n* = 4). L‐N) Colon expression of ZO‐1 and Occludin assayed by western blot and quantitation using Image J software (*n* = 3). O‐Q) Colon expression of ZO‐1 and Occludin assayed by immunohistochemistry and calculation of integrated option density using Image Pro Plus software (scale bar, 50 µm, *n* = 3). Data were shown as mean ± SEM. Statistical analysis was performed by Kruskal‐Wallis test (B) or one‐way ANOVA with Dunnett's post‐test (C‐K, M‐N, and P‐Q). *p < 0.05; **p < 0.01; ***p < 0.001; ns, no significance.

To investigate whether *B. intestinihominis* can alleviate hyperglycemia and liver metabolic disorders, HFD/STZ mice were treated with live *B. intestinihominis* (LBI) and heat‐killed *B. intestinihominis* (KBI) by daily oral gavage for 6 weeks (**Figure** **2**A). Oral administration of LBI elevated *B. intestinihominis* level in the gut of HFD/STZ mice. The abundance of *B. intestinihominis* remained unaltered in KBI‐treated HFD/STZ mice (Figure 2B). Body weight did not differ among the chow, HFD/STZ, LBI, and KBI groups (Figure S5A,B, Supporting Information). Oral administration of LBI alleviated fasting blood glucose levels, serum insulin levels, the HOMA‐IR index, and OGTT and ITT results (Figure 2C–G). Furthermore, LBI treatment also reduced liver weight and the liver weight/body weight ratio and improved dyslipidemia (TG and TC) (Figure 2H; Figure S5C–E, Supporting Information). The plasma ALT and AST levels were significantly decreased by LBI (Figure S5F,G, Supporting Information). The NAS was higher in HFD/STZ mice compared to chow mice, indicating that HFD/STZ mice had liver steatosis and injury. Oral administration of LBI alleviated liver steatosis and injury in HFD/STZ mice (Figure 2I). However, KBI treatment had no effects on serum insulin levels, the OGTT and ITT results, liver weight, dyslipidemia, liver steatosis and injury, ALT and AST levels (Figure 2D,F–I; Figure S5A–G, Supporting Information). These findings indicate that LBI is beneficial and protective against hyperglycemia and liver metabolic disorders in T2D mice.

### 2.3 *B. Intestinihomini* Prevented Leaky Gut and Metabolic Endotoxaemia

Given that T2D is associated with leaky gut via the gut‐living axis,^[^ ^15^, ^26^ ^]^ we examined the effect of LBI on gut barrier functions in an HFD/STZ‐induced mouse model. Oral administration of LBI significantly decreased lipopolysaccharide (LPS) levels in serum (Figure 2J). Histological staining of colonic tissues revealed that, compared with HFD/STZ mice, LBI was able to maintain gut epithelial barrier function (Figure 2K). The epithelial expression of Occludin and ZO‐1 was significantly downregulated in HFD/STZ mice, whereas this suppression was reversed by the administration of LBI (Figure 2L–Q).

### 2.4 *B. Intestinihomini*‐Derived Acetate Mirrored the Favourable Effects

**Figure 3:**
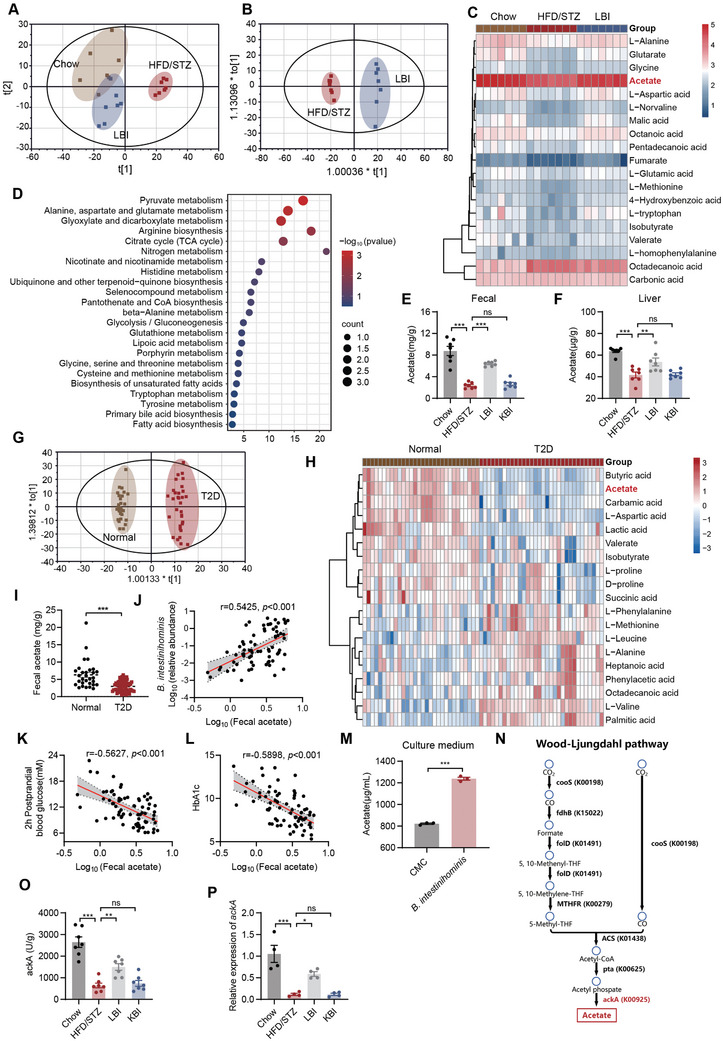
Metabolomic identified the *B. intestinihominis*‐derived metabolites. A) PCA score plots for discriminating the fecal metabolome from chow, HFD/STZ, and LBI groups (*n* = 7). B) OPLS‐DA analysis of metabolic profiles in HFD/STZ and LBI groups (*n* = 7). C) Heatmaps of the differential metabolites that were altered by HFD/STZ compared with LBI‐treated mice (*n* = 7). D) Metabolic pathways in the HFD/STZ versus LBI groups. E) Acetate levels in fecal (*n* = 7). F) Acetate levels in the liver (*n* = 7). G) OPLS‐DA analysis of metabolic profiles in healthy subjects (*n* = 30) and T2D patients (*n* = 32). H) Heatmaps of the differential metabolites that were altered by healthy subjects (*n* = 30) compared with T2D patients (*n* = 32). I) The levels of acetate from T2D patients (*n* = 71) and healthy subjects (*n* = 30). J) Pearson's analysis of the correlations between the abundance of *B. intestinihominis* and acetate levels. K‐L) Pearson's analysis of the correlations between the levels of acetate and 2hPBG (K) and HbA1c (L), respectively. M) Acetate levels in the supernatant after 48 h of incubation with *B. intestinihominis* (*n* = 3). N) Wood‐Ljundahl pathway. O) The viability of ackA in fecal (*n* = 7). P) Relative expression of ackA in fecal (*n* = 4). Data were shown as mean ± SEM. Statistical analysis was performed by one‐way ANOVA with Dunnett's post‐test (E‐F and O‐P), two‐tailed Student's t‐test (M), or Mann‐Whitney U test (I). *p < 0.05; **p < 0.01; ***p < 0.001; ns, no significance.

We performed untargeted metabolomics to evaluate the differential fecal metabolic profiles (**Figure** **3**A,B; Figure S6A–C, Supporting Information). Twenty differential metabolites between the chow and HFD/STZ groups were identified by a variable importance in projection (VIP) ≥ 1.0 and a false discovery rate (FDR) of *p* < 0.05 (Table S3, Supporting Information). Oral administration of LBI significantly regulated 19 metabolites compared with HFD/STZ mice (Figure 3C; Table S4, Supporting Information). Notably, acetate was the most abundant and significantly enriched metabolite, correlated with most of the annotated top 5 KEGG pathways, such as pyruvate metabolism, glyoxylate, and dicarboxylate metabolism (Figure 3D; Figure S6D, Supporting Information). Acetate is the end product of microbial fermentation in the gut.^[^ ^27^ ^]^ Previous reports have revealed the benefits of acetate in attenuating metabolic diseases.^[^ ^28^, ^29^, ^30^ ^]^ In this study, the fecal and liver tissue levels of acetate were significantly decreased in the HFD/STZ mice, while treatment with LBI significantly upregulated using GC‐MS‐Based targeted metabolomics (Figure 3E,F; Figure S7A,B, Supporting Information). Additionally, the fecal levels of acetate were inversely associated with multiple metabolic parameters (Figure S7C–H, Supporting Information).

Furthermore, we evaluated the differences in fecal metabolic profiles using untargeted metabolomics between healthy subjects and T2D patients and identified 19 differential metabolites (Figure 3G,H; Figure S8A–C and Table S5, Supporting Information). Of note, the acetate levels were significantly decreased in the fecal samples from T2D patients, and acetate levels in T2D patients were also significantly lower than those in healthy subjects using targeted metabolomics (Figure 3I). Acetate levels were significantly correlated with *B. intestinihominis*, 2hPBG, and HbA1c (Figure 3J–L).

After 48 h of in vitro incubation with *B. intestinihomini*, acetate significantly accumulated in the supernatant compared with the vector control (Figure 3M; Figure S7I, Supporting Information). The Wood‐Ljundahl pathway is the main pathway generating acetate by gut microbes.^[^ ^31^ ^]^ Acetate is synthesized via 2 branches: 1) the C1‐body branch via the reduction of CO_2_ to formate and 2) the carbon monoxide branch via the reduction of CO_2_ to CO, which further combines with a methyl group to produce acetyl‐CoA. Acetyl‐CoA is catalyzed by the enzymes phosphate acetyltransferase (pta) and acetate kinase (ackA), with ackA controlling the ultimate production of acetate (Figure 3N).^[^ ^27^, ^32^ ^]^ We observed that the activity and expression of ackA were significantly increased after LBI administration (Figure 3O,P). Therefore, the decreased abundance of *B. intestinihominis* is more likely to lead to a reduction in acetate production.

**Figure 4:**
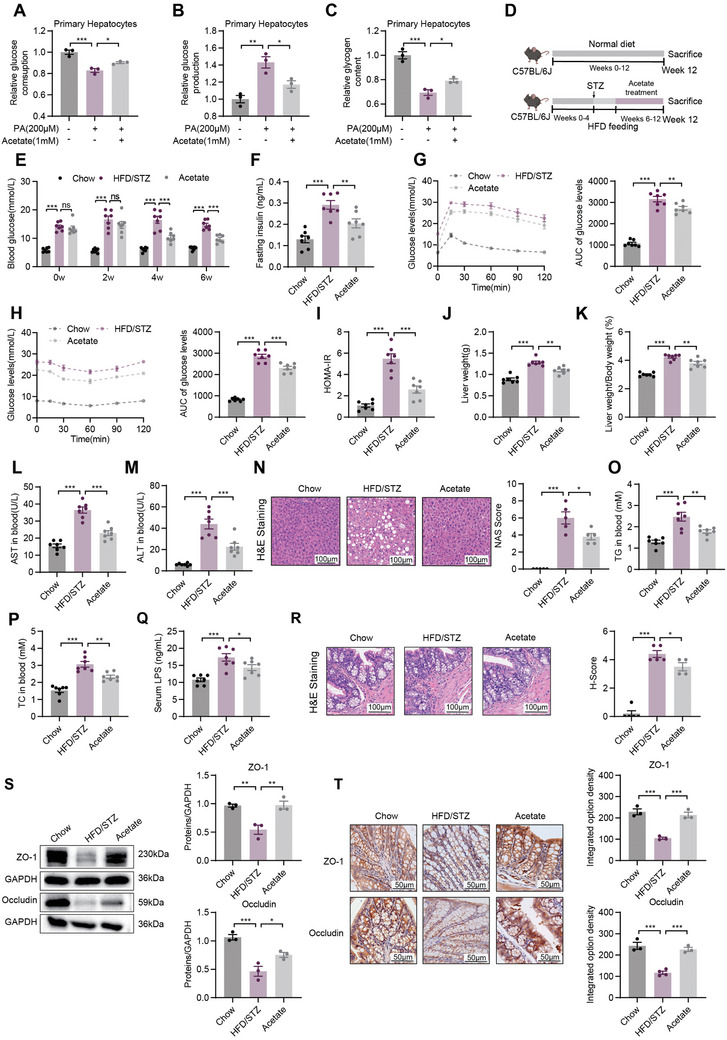
Acetate attenuated metabolic disorder and gut barrier dysfunction in HFD/STZ mice. A‐C) The consumption A), gluconeogenesis B), and the glycogen content C)were detected (*n* = 3) in primary hepatocytes. D) A schematic diagram showing the procedure of HFD/STZ mice treated with acetate. E) Fasting blood glucose levels at 2, 4, and 6 weeks (*n* = 7). F) Insulin levels (*n* = 7). G‐H) OGTT G) and ITT H) with AUC (*n* = 7). I) HOMA‐IR index (*n* = 7). J) Liver weight (*n* = 7). K) Liver weight/body weight ratio (*n* = 7). L‐M) AST and ALT levels in the blood (*n* = 7). N) Representative photomicrographs of liver H&E staining and histological scores (scale bar, 100 µm, *n* = 5). O‐P) TG and TC levels in the blood (*n* = 7). Q) The serum LPS levels (*n* = 7). R) Representative photomicrographs of colon H&E staining and histological scores (scale bar, 100 µm, *n* = 4–5). S) Colon expression of ZO‐1 and Occludin assayed by western blot and quantitation using Image J software (*n* = 3). T) Colon expression of ZO‐1 and Occludin assayed by immunohistochemistry and calculation of integrated option density using Image Pro Plus software (scale bar, 50 µm, *n* = 3–4). Data were shown as mean ± SEM. Statistical analysis was performed by one‐way ANOVA with Dunnett's post‐test. *p < 0.05; **p < 0.01; ***p < 0.001; ns, no significance.

Incubation with acetate for 24 h significantly decreased hepatic glucose levels by inhibiting gluconeogenesis and increasing glycogen synthesis in PA‐induced primary hepatocytes, HepG2 cells, and HL‐7702 cells (**Figure** **4**A–C; Figure S9A–H, Supporting Information). The administration of acetate decreased fasting blood glucose levels, insulin levels, and the HOMA‐IR index, and improved OGTT and ITT results in HFD/STZ mice (Figure 4D–I; Figure S10, Supporting Information). It significantly ameliorated liver metabolic disorders, as indicated by liver weight, liver weight/body weight ratio, pathological alterations, lipid content (TG and TC), and AST and ALT levels (Figure 4J–P). Acetate decreased serum LPS levels and improved the gut barrier structure in HFD/STZ mice (Figure 4Q–T). Taken together, these results indicate that acetate was the functional metabolite of *B. intestinihominis* that ameliorated hyperglycemia and liver metabolic disorders to combat T2D.

### 2.5 *B. Intestinihominis*‐Derived Acetate Activated FGF21 Gene Transcription Through HDAC9

**Figure 5:**
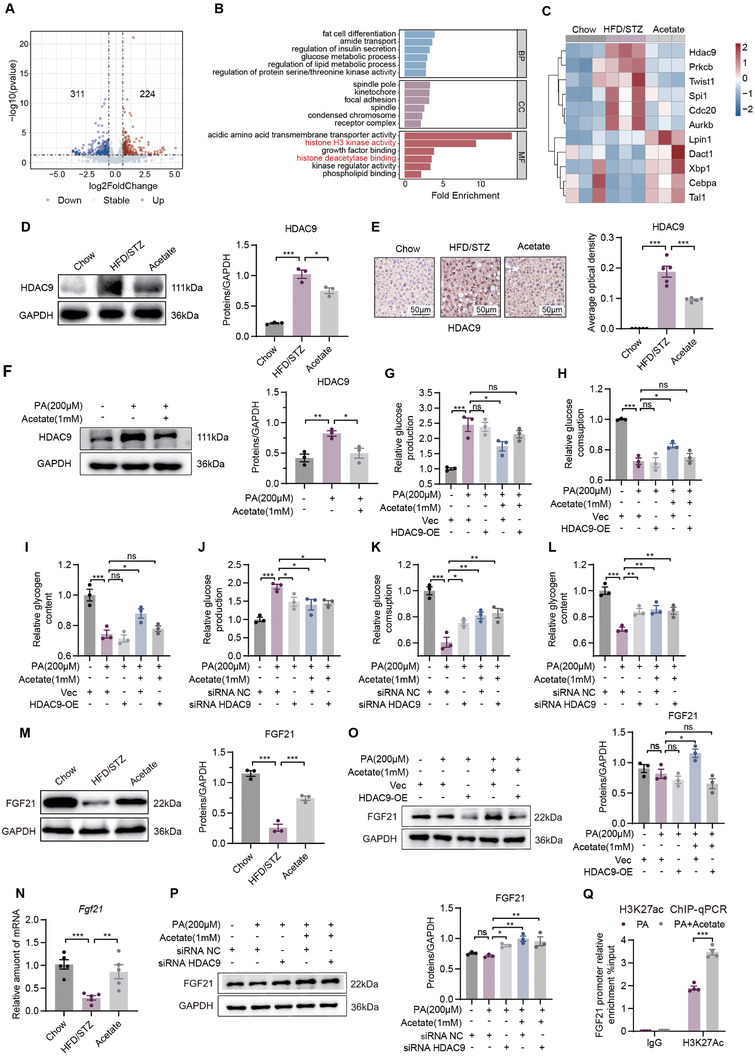
Acetate activated FGF21 gene transcription through down‐regulation of HDAC9. A) Volcano plot shows the number of differential genes downregulated or upregulated by acetate (*n* = 3). B) Gene ontology analysis in the HFD/STZ versus LBI groups (*n* = 3). C) Heatmaps of the differential gene related to histone deacetylation terms (*n* = 3). D) Liver expression of HDAC9 assayed by western blot and quantitation using Image J software (*n* = 3). E) Liver expression of HDAC9 assayed by immunohistochemistry and calculation of average option density using Image Pro Plus software (*n* = 5). F) Western blotting analysis of HDAC9 expression in HepG2 cells (*n* =  3). G–I) Detection of the gluconeogenesis G), glucose consumption H), and glycogen content I) after overexpression of HDAC9 (*n* = 3). J‐L) Detection of the gluconeogenesis (J), glucose consumption (K), and the glycogen content L) after knocking down HDAC9 (*n* = 3). M) Liver expression of FGF21 assayed by western blot and quantitation using Image J software (*n* = 3). N) Relative mRNA expression of FGF21 in the liver (*n* = 5). O) Overexpression of HDAC9 to detect the expression of FGF21 in HepG2 cells (*n* = 3). P) Detection of FGF21 expression in HepG2 cells after knocking down HDAC9 (*n* = 3). Q) Detection of H3K27ac enrichment in the FGF21 promoter region using ChIP‐qPCR (*n* = 4). Data were shown as mean ± SEM. Statistical analysis was performed by one‐way ANOVA with Dunnett's post‐test (D‐P) or two‐tailed Student's t‐test (Q). *p < 0.05; **p < 0.01; ***p < 0.001; ns, no significance.

RNA sequencing was conducted on liver tissues from the chow, HFD/STZ, and acetate treatment groups (Figure S11A, Supporting Information). HFD/STZ treatment resulted in the upregulation of 1434 genes and the downregulation of 513 genes (Figure S11B, Supporting Information). Oral administration of acetate significantly upregulated 224 genes and downregulated 311 genes (**Figure** **5**A). The differential gene ontology (GO) analysis showed the mechanism of ameliorated hyperglycemia and liver metabolic disorders was related to histone deacetylation, and *Hdac9* was significantly downregulated by acetate (Figure 5B,C; Figure S11C, Supporting Information). The downregulated mRNA and protein levels of HDAC9 in the liver and HepG2 cells were confirmed after acetate treatment (Figure 5D–F; Figure S12A,B, Supporting Information). Besides, molecule docking showed that acetate has of potential to chelate the zinc ion in HDAC9 using the Glide algorithm (Figure S12C, Supporting Information).

Next, we investigated the role of liver HDAC9 in the process by which acetate regulates metabolic disorders. The overexpression of HDAC9 abolished the protective effect of acetate in PA‐induced HepG2 cells (Figure 5G–I; Figure S12D, Supporting Information), but HDAC9 siRNA significantly reversed gluconeogenesis, glucose consumption and glycogen synthesis in PA‐induced HepG2 cells (Figure 5J–L; Figure S12E, Supporting Information).

Fibroblast growth factor 21 (FGF21) is abundantly expressed in the liver and is closely associated with metabolic diseases.^[^ ^33^, ^34^ ^]^ Previous studies have reported that HDACs can upregulate the expression of FGF21 by promoting histone acetylation in the FGF21 promoter region.^[^ ^35^, ^36^ ^]^ In this study, *B. intestinihominis* and its derived acetate significantly upregulated FGF21 levels in HFD/STZ mice (Figure 5M,N; Figure S12F, Supporting Information). In PA‐induced HepG2 cells, we found that overexpression of HDAC9 abolished the upregulation of FGF21 by acetate (Figure 5O). Similarly, knockdown of HDAC9 upregulated FGF21 (Figure 5P). More importantly, ChIP‐qPCR analysis revealed that acetate upregulated histone H3K27 acetylation at the FGF21 promoter (Figure 5Q). Overall, these data suggested that *B. intestinihominis*‐derived acetate upregulated histone acetylation of the FGF21 promoter region and promoted the transcription of FGF21 by inhibiting the expression of HDAC9.

### 2.6 Modulators that Ameliorated Hyperglycemia and Liver Metabolic Disorders were Screened by Promoting the Growth of *B. Intestinihominis*

Previous studies have shown that herbs can alleviate metabolic diseases by modulating the gut microbiota.^[^ ^37^, ^38^ ^]^ We screened 48 components from T2D‐related herbs (Gegen Qinlian decoction) for promotion of *B. intestinihominis* (Table S6, Supporting Information).^[^ ^39^, ^40^ ^]^ Puerarin had the strongest ability to facilitate the growth of *B. intestinihominis* (Figure S13, Supporting Information). Oral administration of puerarin can restore the abundance of *B. intestinihominis*, and increase the acetate levels (Figure S14A–C, Supporting Information). Puerarin significantly ameliorated glucose homeostasis, liver steatosis, and injury in HFD/STZ mice. (Figure S14D–Q, Supporting Information). The expression of HDAC9 was downregulated and that of FGF21 was upregulated after puerarin treatment (Figure S14R, Supporting Information).

**Figure 6:**
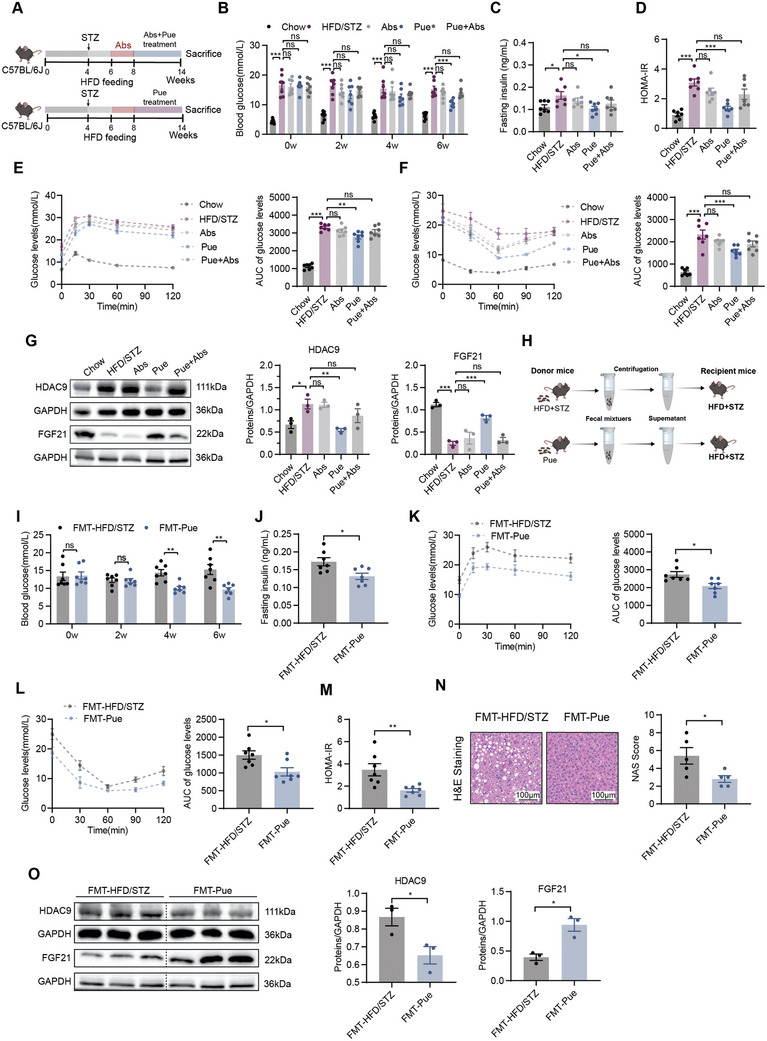
Puerarin attenuated hyperglycemia and liver metabolic disorder in HFD/STZ mice in a gut microbiota‐dependent manner. A) Schematic diagram for antibiotic treatment. B) Fasting blood glucose levels at 2, 4, and 6 weeks for antibiotic treatment (*n* = 7). C) Insulin levels for antibiotic treatment (*n* = 7). D) HOMA‐IR index for antibiotic treatment (*n* = 7). E‐F) OGTT (E) and ITT (F) with AUC (*n* = 7). G) Liver expression of HDAC9 and FGF21 in antibiotic‐treated HFD/STZ mice assayed by western blot and quantitation using Image J software (*n* = 3). H) Experimental design diagram for fecal microbiota transplantation. I) Fasting blood glucose levels at 2, 4, and 6 weeks after fecal microbiota transplantation (*n* = 7). J) Insulin levels for fecal microbiota transplantation (*n* = 7). K‐L) OGTT (K) and ITT (L) with AUC (*n* = 7). M) HOMA‐IR index for fecal microbiota transplantation (*n* = 7). N) Representative photomicrographs of liver H&E staining and histological scores after fecal microbiota transplantation (scale bar, 100 µm, *n* = 5). O) Liver expression of HDAC9 and FGF21 after fecal microbiota transplantation assayed by western blot and quantitation using Image J software (*n* = 3). Data were shown as mean ± SEM. Statistical analysis was performed by one‐way ANOVA with Dunnett's post‐test (B‐G) or two‐tailed Student's t‐test (I‐O). *p < 0.05; **p < 0.01; ***p < 0.001; ns, no significance.

To investigate whether the metabolic protective effects of puerarin are dependent on the presence of the gut microbiota, we treated HFD/STZ mice with a cocktail of antibiotics (**Figure** **6**A). These results indicated that the improvement in hyperglycemia and liver metabolic disorders caused by puerarin was abolished under antibiotic cocktail intervention (Figure 6B–F; Figure S15A–G, Supporting Information). The reversal of HDAC9 and FGF21 expression was also blocked (Figure 6G).

Moreover, we transferred the microbiota from puerarin‐treated HFD/STZ mice to conventional recipient mice with HFD/STZ. After 6 weeks of colonization, puerarin receivers (FMT‐Pue) significantly improved hyperglycemia, liver steatosis, and injury compared with HFD/STZ receivers (FMT‐HFD/STZ, Figure 6H–N; Figure S16A–F, Supporting Information). The changes in the expression of HDAC9 and FGF21 in puerarin receivers were reversed (Figure 6O). Together, these results demonstrated that puerarin, as a prebiotic agent protected against T2D in a gut microbiota‐dependent manner.

### 2.7 *B. Intestinihominis*, Acetate and Puerarin Alleviated Hyperglycemia and Liver Metabolic Disorders in db/db Mice

**Figure 7:**
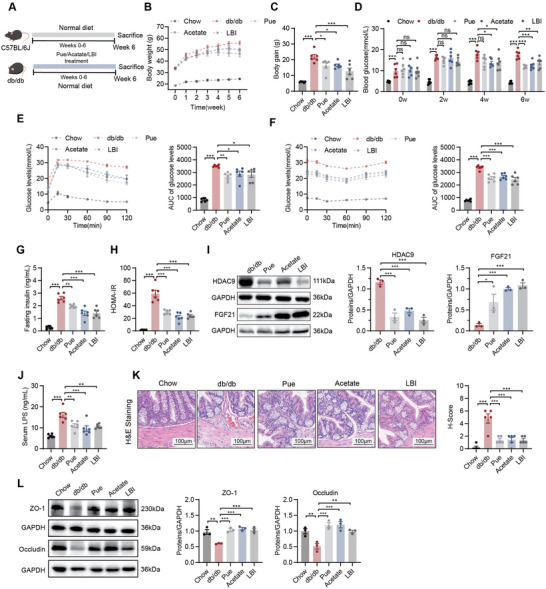
LBI, acetate, and puerarin attenuated hyperglycemia and liver metabolic disorder in db/db mice. A) A schematic diagram showing the procedure of db/db mice treated with LBI, acetate, and puerarin. B) Body weight curve (*n* = 6). C) Body weight gain (*n* = 6). D) Fasting blood glucose levels at 2, 4, and 6 weeks (*n* = 6). E‐F) OGTT (E) and ITT (F) with AUC (*n* = 6). G) Insulin levels (*n* = 6). H) HOMA‐IR index (*n* = 6). I) Liver expression of HDAC9 and FGF21 assayed by western blot and quantitation using Image J software (*n* = 3). J) The serum LPS levels (*n* = 6). K) Representative photomicrographs of colon H&E staining and histological scores (scale bar, 100 µm, *n* = 5). L) Colon expression of ZO‐1 and Occludin assayed by western blot and quantitation using Image J software (*n* = 3). Data were shown as mean ± SEM. Statistical analysis was performed by one‐way ANOVA with Dunnett's post‐test (C‐L). *p < 0.05; **p < 0.01; ***p < 0.001; ns, no significance.

As expected, administration of LBI, acetate, or puerarin improved body weight, fasting blood glucose, OGTT, ITT, insulin levels, and the HOMA‐IR index in db/db mice (**Figure** **7**A–H). It also significantly ameliorated liver steatosis and injury (Figure S17A–F, Suppporting Information). LBI, acetate, and puerarin treatment significantly decreased HDAC9 and increased FGF21 expression (Figure 7I). Gut epithelial barrier function was also ameliorated (Figure 7J–L).

## 3 Discussion

The gut microbiome plays an increasingly important role in the onset and progression of obesity, T2D, MAFLD, and other hallmarks of metabolic syndromes.^[^ ^16^, ^41^ ^]^ The administration of probiotics or the inhibition of pathogenic gut microbes represent potential alternative therapeutic strategies for T2D management.^[^ ^26^, ^29^ ^]^ In our study, we found that the abundance of *B. intestinihominis* was decreased in HFD/STZ mice and T2D patients, and was negatively correlated with 2hPBG and HbA1c. *B. intestinihominis* is a gram‐negative bacterium that was first isolated from the feces of a healthy human. *B. intestinihominis* was associated with cerebral small vessel disease^[^ ^42^ ^]^ and metastatic renal cell carcinoma.^[^ ^43^ ^]^ Oral administration of *B. intestinihominis* resulted in accumulation in the colon and promoted the infiltration of IFN‐γ‐producing γδT cells in cancer lesions.^[^ ^44^ ^]^ In general, these studies raised the possibility that *B. intestinihominis* might be important for the establishment of beneficial gut microbiota. However, there are no reports on the role of *B. intestinihominis* in T2D. We revealed that oral administration of LBI ameliorated hyperglycemia and liver metabolic disorders in HFD/STZ and db/db mice.

We then investigated how *B. intestinihominis* ameliorated hyperglycemia and liver metabolic disorders. The present study revealed increased levels of acetate in the feces and liver after LBI treatment, suggesting that acetate may mediate most of the effects of *B. intestinihominis*. Acetate is recognized as a functional metabolite derived from bacteria.^[^ ^27^ ^]^ Acetate has been reported to improve fasting blood glucose levels and insulin sensitivity.^[^ ^28^, ^29^ ^]^ In addition, acetate was shown to support metabolic improvements in MAFLD by activating the G‐protein‐coupled receptor 43 (GPR43) pathway.^[^ ^45^ ^]^ A recent study revealed that *Lactobacillus reuteri‐*derived acetate could inhibit histone deacetylase activity and regulate Group 3 innate lymphoid cells in hepatocellular carcinoma.^[^ ^46^ ^]^ In this study, we found that treatment with acetate can inhibit HDAC9 in the liver and hepatocytes. HDAC9 is a member of the class IIa HDAC family and acts as an epigenetic regulator that deacetylates nucleosome histones and suppresses gene expression. Accumulating evidence has linked FGF21 with multiple metabolic activities.^[^ ^33^, ^34^ ^]^ Thus, we hypothesized that HDAC9 can inhibit FGF21 expression through promoter histone hypoacetylation. The present study verified that acetate enhanced FGF21 expression through inhibition of HDAC9 to increase the H3K27ac modification. Consistently, LBI treatment also inhibits HDAC9 to promote FGF21 expression. Accordingly, these findings provide compelling evidence to support the assertion that *B. intestinihominis* has ameliorative effects on metabolic disorders through the production of acetate.

Active components from medicinal plants have been demonstrated to alleviate metabolic disorders by modulation of gut microbiota.^[^ ^47^ ^]^ Thus, we hypothesized that certain active compounds could attenuate metabolic disorders by promoting *B. intestinihominis*. In this study, we found that puerarin significantly enriched the abundance of *B. intestinihominis* via in vitro fermentation, and this finding was confirmed in puerarin‐treated mice. A recent study revealed that puerarin injection modulates an extremely novel brain‐gut signal that regulates fat absorption in the intestine.^[^ ^48^ ^]^ In addition, as the absolute bioavailability of puerarin is less than 7%, it can play an essential role in modulating the gut microbial ecosystem after oral administration.^[^ ^49^, ^50^ ^]^ For example, puerarin alleviates atherosclerosis by inhibiting the trimethylamine of *Prevotella copr*.^[^ ^51^ ^]^ Based on these findings, we additionally verified that the effect of puerarin on hyperglycemia and liver metabolic disorders was dependent on the gut microbiota in both antibiotic‐treated mice and FMT models.

## 4 Conclusion

**Figure 8:**
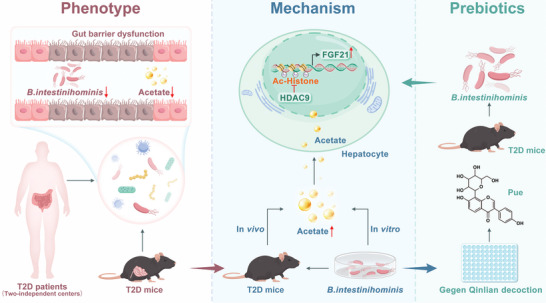
Schematic illustration of the proposed underlying mechanism by how *B. intestinihominis* ameliorated hyperglycemia and liver metabolic disorders in T2D.

In summary, the abundance of *B. intestinihominis* was significantly reduced in the fecal of T2D patients from 2 independent centers. Oral administration of live *B. intestinihominis* attenuated hyperglycemia and liver metabolic disorders in T2D. *B. intestinihominis*‐derived acetate inhibited HDAC9 to increase the FGF21 expression. The screened puerarin in a gut microbiota‐dependent manner protected against T2D by promoting the growth of *B. intestinihominis* (**Figure** **8**). Our study highlights the gut commensal *B. intestinihominis* as a probiotic and puerarin as a prebiotic agent for the alleviation of T2D.

## 5 Experimental Section

### Human Samples

Fecal samples from 31 T2D patients and 30 healthy subjects were collected from Nanjing Hospital of Chinese Medicine affiliated with the Nanjing University of Chinese Medicine for shotgun metagenomics as a microbiota discovery set (Table S1, Supporting Information). Fecal samples from 207 T2D patients and 103 healthy subjects were collected from Nanjing Hospital of Chinese Medicine affiliated with Nanjing University of Chinese Medicine and the First Affiliated Hospital of Zhengzhou University for microbiota validation sets (Table S2, Supporting Information). Patients were excluded if they had gastrointestinal pathology, cancer, autoimmune disorders, infectious diseases, or had used antibiotics in the previous 3 months. All procedures were approved by the hospital's medical ethics committee (No: KY2023294) and followed the tenets of the Declaration of Helsinki. The collection, preservation, and processing of the fecal samples were conducted according to a previous protocol.^[^ ^52^ ^]^

### Animal Study

Animal experiments were conducted in accordance with the Guidelines for the Animal Experiment Center of China Pharmaceutical University (Nanjing, China), and the protocols were approved by the Animal Care and Utilization Committee of this institution (No: 202 002 001). The male C57BL/6J (SPF grade, 6 weeks old, 18–20 g) and 8‐week‐old db/db mice were purchased from Vital River Laboratory Animal Technology Co. Ltd. (Beijing, China) and GemPharmatech Co. Ltd. (Nanjing, China), respectively. All animals were housed in SPF conditions on a standard 12‐h light‐dark cycle with free access to food and water at a controlled temperature (25 ± 2 °C).

The C57BL/6J mice were randomly divided into the standard chow diet (Jiangsu synergetic biology, 1 010 039) group and the HFD (60% fat, Research Diets, D12492) group. After exposure for 4 weeks to the respective diets, the HFD‐fed mice were injected intraperitoneally with 40 mg k^−1^g STZ solution after overnight fasting for 5 consecutive days. The chow group received the vehicle only. After 7 days, the HFD/STZ mice with fasting blood glucose levels ≥ 11.1 mM were considered T2D mice. T2D mice were maintained with HFD feeding for 6 weeks, and other mice were fed with a standard chow diet. In assays involving *B. intestinihomini*, HFD/STZ mice in the LBI group were treated daily with live *B. intestinihomini*. Mice in the KBI group were given heat‐killed *B. intestinihomini*. Treatments were continued for 6 weeks. In experiments with acetate, HFD/STZ mice were divided into 3 groups. Acetate‐treated groups were given acetate (200 mg k^−1^g) daily by gavage. Treatments were continued for 6 weeks. Similarly, HFD/STZ mice were grouped and subsequently treated with puerarin (200 mg k^−1^g). For in vivo antibiotic treatment, HFD/STZ mice were supplemented with either 0.5% CMCNa (solvent) or puerarin at 200 mg k^−1^g in the absence or presence of antibiotics [vancomycin (50 mg k^−1^g), neomycin sulfate (100 mg k^−1^g), metronidazole (100 mg k^−1^g), and ampicillin (100 mg k^−1^g)] daily by gavage. A fecal transplant was performed based on an established protocol.^[^ ^53^ ^]^ Briefly, stools from puerarin‐treated mice or HFD/STZ mice were collected, snap‐frozen, and stored at −80 °C. HFD/STZ mice were pre‐treated with combined antibiotics daily by gavage until 2 weeks. After Abs treatment, the recipient mice were treated daily with transplant material from either puerarin‐treated mice or HFD/STZ mice. The stools (100 mg) from donor mice were diluted with saline and homogenized for 1 min using a vortex to achieve a liquid slurry and then centrifuged at 800 *g* for 3 min to remove particulate matter to facilitate administration. Oral gavage with fecal transplant material was conducted daily throughout the 6‐week experiment. In experiments with db/db mice, the wild‐type (WT) littermates were used as controls. The db/db mice were divided into 4 groups, the LBI group was treated daily with live *B. intestinihomini* (0.2 mL/10 g), acetate‐treated groups and puerarin‐treated groups were given acetate (200 mg k^−1^g), and puerarin (200 mg k^−1^g), respectively. Treatments were continued by gavage daily for 6 weeks.

The body weight of the mice was measured on a scale every week, and fasting blood glucose was examined every 2 weeks. OGTT and ITT were conducted before mice were sacrificed. Blood samples were collected at the end of the experiments. The liver, fat, pancreas, muscle, cecum, intestines colon, and fecal were quickly frozen and stored at −80  °C. The partial tissues of the colon and liver were immediately stored in 4% paraformaldehyde for histopathological examination. The remaining liver and colon, as well as other tissues such as fat, pancreas, muscle, and cecum, were quickly frozen and stored at −80  °C.

### 16S rRNA Bacteria Gene Sequencing

Bacterial DNA was extracted from fecal samples using the MagPure Soil DNA LQ Kit (MAGEN, Guangzhou, China), and the DNA concentration and purity were assessed with a Qubit 2.0 fluorometer. The hypervariable region V3‐V4 of the bacterial 16 S rRNA gene was amplified using primer pairs 343F (5′‐TACGGRAGGCAGCAG‐3′) and 798R (5′‐AGGGTATCTAATCCT‐3′). The PCR amplicons were purified with AMPure XP beads. DNA libraries were validated by Agilent 2100 Bioanalyzer. Sequencing was conducted on a HiSeq PE250 platform (Illumina). Quality filtering on the raw reads was performed under specific filtering conditions to obtain the high‐quality clean tags according to the Cutadapt (v.2.6). Chimeric sequences were filtered using Readfq (v1.0). After de‐replication using DADA2, the feature table and feature sequence were obtained. The alpha diversity and beta diversity were analyzed by QIIME2. LEfSe (v.1.1.2) was performed to detect differential abundant taxa across groups using LDA score (log 10)>4.

### Metagenomic Sequencing

Fecal samples from 31 T2D patients and 30 healthy subjects were collected for metagenomic profiling (Table S1, Supporting Information). Metagenomic sequencing was carried out by LC‐Bio Technology Co., Ltd (Hang Zhou, China). In brief, metagenome libraries were sequenced on an Illumina NovaSeq 6000 platform with PE150 (Hangzhou, China). The adaptor and low‐quality reads were trimmed by Fastp software (v0.23.4) using a sliding‐window algorithm, and the remaining reads were subjected to denovo assembly for each sample using MEGAHIT (v1.2.9). All coding regions (CDS) of metagenomic contigs were predicted by MetaGeneMark (v3.26), and the unigenes were obtained by clustering the CDS sequences of all samples using MMseq2 (v15‐6f452. After that, DIAMOND (v0.9.14) was used to perform a taxonomic assessment of the microbiota based on the NR database.

### Fecal DNA Extraction and Quantification of Bacteria

Fecal DNA was extracted using the TIANamp Stool DNA Kit (TIANGEN Biotech Co. Ltd., DP328) according to the manufacturer's protocol, and the concentration was measured by a Nano‐100 micro‐spectrophotometer (Hangzhou AllSheng Instruments Co. Ltd.). qPCR assays were performed using the AceQ qPCR SYBR Green Master Mix (Vazyme Biotech, Q111) by the Light‐Cycler 480 PCR System (Roche Diagnostics). The primer used to amplify the bacteria is described in Table S7 (Supporting Information).

### Culture and Preparation of *B. Intestinihomini*

*B. Intestinihomini* (strain: DSM 21 032) was purchased from the German Collection of Microorganisms and cultivated in Chopped Meat Carbohydrate Broth(CMC) medium. An anaerobic chamber containing 10% CO_2_, 10% H_2_, and 80% N_2_ was used for all anaerobic microbiological works. Cultures were collected in the log phase and diluted with sterile phosphate‐buffered saline (PBS) to 2 × 10^8^ colony‐forming units/mL for gavage. For the KBI trials, *B. intestinihomini* were heat‐killed at 121 °C (treatment duration, 20 min). Mice were orally administered either LBI or KBI (0.2 mL/10 g) daily.

### *B. Intestinihomini* Growth Modulators using In Vitro Screening

The cultivated *B. intestinihomini* was seeded in 96‐well plates under the initial OD_600_ value of ≈0.2, and treated with 48 active natural compounds (50 µM, Table S6, Supporting Information). Then, the OD_600_ value was recorded after co‐cultivation at different times of 0, 6, 12, 24, 36, 48, 60, and 72 h using a microplate reader. Modulators were screened through observation of the effects on the growth curve of *B. intestinihomini* in vitro.

### Cell Culture and Treatments

HepG2 cells (ATCC, HB‐8065) and HL‐7702 cells (KeyGen Biotech, KG063,) were in Dulbecco's modified eagle's medium supplemented with penicillin (100 U mL^−1^), streptomycin (100 µg mL^−1^), and 10% (v/v) fetal bovine serum (FBS) under 37 °C, 5% CO_2_. The mouse primary hepatocytes were isolated and cultured according to a previous report.^[^ ^54^ ^]^ For drug treatment, the HepG2, HL‐7702, and primary hepatocytes were stimulated with palmitic acid (PA, 200 µM, Sigma, P5585), acetate (1 mM, yuanye Bio‐Technology, S30901) for 24 h.

### mRNA Isolation and qPCR

Total mRNA was extracted using a FreeZol Reagent RNA Isolation Kit (Vazyme Biotech, R711) according to the manufacturer's instructions. Total RNA was reverse transcribed by a HiScript II Q RT SuperMix for qPCR (+gDNA wiper, R233‐01, Vazyme Biotech) for qPCR according to the manufacturer's instructions. Quantitative qPCR was carried out by the Light‐Cycler 480 PCR System (Roche Diagnostics) using AceQ qPCR SYBR Green Master Mix (Vazyme Biotech, Q111). Primer sets for genes are presented in Table S7 (Supporting Information).

### Metabolic Tolerance Tests

Oral Glucose Tolerance test (OGTT) and Insulin Tolerance test (ITT) were performed on each group of mice. After an overnight fast, mice were gavaged with 0.5 g kg^−1^ glucose. After fasting for 4 h, mice were injected with 0.5 U kg^−1^ insulin by intraperitoneal injection. Tail blood was drawn to determine the glucose levels before administration (time 0) and 15, 30, 60, 90, and 120 min after administration. The interval between 2 experiments is greater than 3 days. The area under the curve (AUC) was calculated to quantify the blood glucose of OGTT and ITT.

### Histopathological and Immunohistochemical Analysis

After euthanasia, the liver and colonic tissues of mice were fixed in 4% paraformaldehyde (Servicebio, G1101). Paraformaldehyde‐fixed, paraffin‐embedded liver tissue slides were stained with H&E. All procedure was performed according to standard protocols followed by microscopy examination. Images of the sections were obtained using a Leica DMi8 fluorescence microscope (Leica, Germany). Under blinded conditions, the liver and colonic sections were scored according to a previous report.^[^ ^55^, ^56^ ^]^ For immunohistochemistry (IHC), paraffin sections were incubated with antibodies specific to HDAC9, Occludin and ZO‐1 [Anti‐HDAC9 (Affinity Biosciences, AF7005), Anti‐Occludin (Cell Signaling Technology, #91 131), Anti‐ZO1 (Abcam, ab221547)]. The average and integrated option density was calculated using Image Pro Plus software.

### Biochemical Analysis

The concentrations of TG, TC, ALT, and AST were analyzed using a spectrophotometer (Bio‐Rad) according to the manufacturer's instructions (Nanjing Jiancheng; TG, A110‐1‐1; TC, A111‐1‐1; ALT, C009‐2‐1; AST, C010‐2‐1). Serum LPS levels were determined by a mouse LPS ELISA kit (Cusabio, CSB‐E13066m) following the manufacturer's instructions. A mouse insulin ELISA kit (Ezassay, MS100/ MS200) was used to detect the insulin levels.

### Glucose Consumption Assay

The HepG2 cells were treated with different compounds for 24 h in the 24‐well plate, the supernatant was collected to be detected using a glucose assay kit (Nanjing Jiancheng, A154‐1‐1). All operations followed the kit instructions.

### Glucose Production Assay

The HepG2 cells were seeded in the 6‐well plate. After treatment, cells were washed twice with phosphate‐buffered saline, cultured in glucose‐free DMEM for 4 h, and replaced with the glucose‐generating medium (20 mM sodium lactate, 2 mM sodium pyruvate, 100 nM insulin) and maintained for 6 h. The supernatant was discarded and the cells were collected in tubes. Measure the glucose content according to the instructions of the kit (Nanjing Jiancheng, A154‐1‐1). Protein was quantified using a BCA assay kit (Beyotime Biotechnology, P0012S) for calibration of glucose levels.

### Glycogen Content

The HepG2 cells were seeded in the 6‐well plate. The HepG2 cells were treated with indicated compounds for 24 h, the supernatant was discarded and the cells were collected in tubes. The glycogen was extracted and detected at the wavelength of 620 nm (Solarbio, BC0345). A BCA assay kit was used to quantify protein content for calibration of glycogen levels. All operations followed the kit instructions.

### Western Blot Analysis

Briefly, protein from cells or mouse liver and colonic tissues were harvested and suspended in a lysis buffer containing protease inhibitor, phosphatase inhibitor, and loading buffer. The lysates were denatured at 95 °C for 10 min. Proteins were separated by 8%–15% SDS‐PAGE and transferred to nitrocellulose (NC) membranes. After blocking with 5% skim milk, the membranes were incubated with corresponding antibodies [Anti‐HDAC9 (Affinity Biosciences, AF7005), Anti‐FGF21 antibody (ABclonal, A10368), Anti‐Occludin (Abcam, ab216327), Anti‐ZO‐1 (Abcam, ab307799), Anti‐GAPDH antibody (Proteintech, 10494‐1‐AP)] overnight at 4 °C. The membranes were washed with TBST and incubated with secondary horseradish peroxidase (HRP)‐conjugated antibodies (HRP‐linked anti‐rabbit IgG (Proteintech, SA00001‐2), HRP‐linked anti‐mouse IgG (Proteintech, RGAM001)) for 1 h at room temperature. The specific protein bands were detected with enhanced chemiluminescence (ECL, Pierce Chemical Co., Ltd).

### Small‐Interfering RNA (siRNA) and Transient Transfection

HepG2 cells were plated in 6‐well plates for 24 h before transfection. siRNA targeting HDAC9 was acquired from Genomeditech (Shanghai, China). The sequences for HDAC9 siRNA1 were listed as follows: 5′‐GAAAGACACUCCAACUAAUTT‐3′ (sense), 5′‐ AUUAGUUGGAGUGUCUUUCTT‐3′ (antisense); the sequences for HDAC9 siRNA2 were 5′‐ CACAUUACCAGGAGCACAATT‐3′ (sense), 5′‐ UUGUGCUCCUGGUAAUGUGTT‐3′ (antisense); the sequences for HDAC9 siRNA3 were 5′‐ GCUCAAUGCUUCGAAUUCATT‐3′ (sense), 5′‐ UGAAUUCGAAGCAUUGAGCTT‐3′ (antisense). The siRNA was transfected using Lipofectamine 3000 transfection reagent according to the manufacturer's protocol.

### Plasmid Transfection

HepG2 cells were seeded in 6‐well plates and cultured in a growth medium to reach 60% confluence. Plasmid encoding HDAC9 vector and control vector were designed and provided by Haixing Biosciences (Suzhou, China). The HDAC9 or control plasmid was transfected using Lipofectamine 3000 transfection reagent according to the manufacturer's protocol.

### Chromatin Immunoprecipitation (ChIP)

The ChIP assay was performed by SimpleChIP Enzymatic Chromatin IP kit (Cell Signaling Technology, #9002) following the manufacturer's protocol. Briefly, HepG2 cells were treated with PA (200 µM) and acetate (1 mM) for 24 h and then fixed with 1% formaldehyde for 10 min, which was stopped by glycine. The cross‐linked chromatin was digested with micrococcal nuclease. An aliquot of each ChIP sample was prepared as input control, while the rest of the DNA was incubated with anti‐Histone H3 (acetyl K27, CST, #8173) or control anti‐IgG antibody overnight at 4 °C. After adding protein G agarose beads for 2 h, chromatin complex was washed by ChIP buffer, and de‐crosslinked by NaCl and proteinase K at 65 °C. Finally, DNA purified through columns was assayed by qPCR using primers for the promoters of FGF21 (Forward: 5′‐ GACTGACCCTCCCATTCAAGATACA‐3′; Reverse: 5′‐ GGAGATGATGGTTAAACTTCTGGG‐3′).

### RNA Sequencing

Total RNA of livers was extracted using TRIzol reagents and RNA quality was determined using an Agilent 2100 Bioanalyser. Sequencing libraries were generated after depleting ribosomal RNA, synthesizing cDNA, and ligating adapters using the DNBSEQ Eukaryotic mRNA library. After cluster generation, the libraries were sequenced using the DNBseq platform. Raw data with adapter sequences or low‐quality sequences were filtered using the SOAPnuke software (v1.5.6). The resulting data was then converted and stored in the fastq format for subsequent analysis. Principal component analysis (PCA) was determined by R package *factoextra*. The differentially expressed genes were carried out by R package *DESeq2* (*p* < 0.05 & FC ≥ 1.5). Gene ontology (GO) analyses were calculated by the R package *clusterProfiler*.

### Non‐Targeted Metabolomics

The protocol for fecal non‐targeted metabolomics was implemented as previously depicted.^[^ ^57^, ^58^ ^]^ Briefly, 30 mg lyophilized fecal was dissolved in 300 µL NaOH solution (1 mM), and homogenized for 5 min. The homogenate was centrifuged, and the 200 µL supernatants were transferred to centrifuge tubes. The precipitate was homogenized for another 5 min with 200 µL of isopropanol, and centrifuged at 13 000 rpm for 10 min. Add the supernatant (167 µL) to the above NaOH solution extracts, and then add 10 µL of internal standard (chloro‐d‐phenylalanine, 1000 µg mL^−1^), 34 µL pyridine and 20 µL propyl chloroformate, shaken for 30 s. Another 20 µL of propyl chloroformate was added again, and samples were shaken for another 30 s. Then 400 µL chloroform and 400 µL NaHCO_3_ solution (50 mM) were added and shaken for 30 s. After the centrifugation, 100 µL of the lower solution was transferred to a sample vial for analysis. Besides10 µL of each sample were pooled to get a quality control sample (QC) that would be tested during the analysis.

The samples were analyzed using an Agilent 7890 chromatograph coupled with a 5977A MS system (Agilent Technologies, USA). Separation was achieved on an Agilent HB‐5MS capillary column (30 m × 0.25 mm, 0.25 µm). The injection volume for untargeted metabolomics was 1 µL. Helium was used as the carrier gas with a constant flow rate of 1 mL mi^−1^n. The optimized temperature gradient was the following: 50 °C held for 3.5 min, then increased at a rate of 9 °C min^−1^ up to 70 °C, 3 °C min^−1^ to 85 °C, 5 °C min^−1^ to 110 °C, 30 °C min^−1^ to 290 °C, and then held there for 8 min. The temperature of the inlet, transfer line, and ion source was set to 250, 290, and 250 °C, respectively. Electron impact ionization (70 eV) at the examined *m*/*z* range of 50–600 was used.

The raw data were converted into mzXML files using MSConvert software and subsequently imported into MS‐DIAL for preprocessing. The parameter settings were as follows: Minimum peak height ≥ 100, Mass slice width: 0.5 Da, Mass accuracy for centroiding: 0.5 Da; Retention Time Tolerance: 0.1 min, EI similarity Tolerance: 70%; frequency filtering criteria: present in at least 50% of one group; and normalization using internal standards. The resulting data set, including retention time, sample names, and peak areas was introduced into the SIMCA‐P 14.0 Software package for multivariate statistical analysis. The significance of each metabolite was analyzed by the Mann‐Whitney‐Wilcoxon test with false discovery rate (FDR) correction via the Benjamini‐Hochberg method. The discrimination of variables was identified by PCA and Orthogonal partial least‐squared discriminant analysis (OPLS‐DA). Differential features were screened by those with variable importance in the projection (VIP) > 1.0 obtained from OPLS‐DA and adjusted *p*‐values less than 0.05. Differential features were identified by NIST library search and confirmed by available references.

### Targeted Quantification of Acetate

Acetate was quantified by Agilent 7890B/5977A GC‐MS equipped with HP‐5MS capillary column (30 m × 0.25 mm × 0.25 µm). A gas using helium as carrier gas (1 mL mi^−1^n). The optimized temperature gradient was the following: 45 °C held for 2 min; rise to 180 °C at 9 °C min^−1^ and held for 5 min; rise to 220 °C at 40 °C min^−1^ and held for 5 min; rise to 240 °C at 40 °C min^−1^ and held for 11.5 min; rise to 280 °C at 40 °C min^−1^ and held for 2 min. The temperature of the inlet, transfer line, and ion source was set to 250, 290, and 250 °C, respectively. The injection volume was 1 µL. Quantitative analysis of acetate and acetic acid ‐d₄ was performed using MRM mode, with ion pairs of 61.0 > 43.1 and 64.0 > 46.1, respectively, and CE values of 15 eV.

For the fecal sample quantitation,^[^ ^59^ ^]^ 50 mg lyophilized fecal was dissolved in 1 mL NaOH solution (5 mM), and 10 µL of internal standard (acetic acid‐d₄, 10 mg mL^−1^), homogenized for 5 min. The homogenate was stored at 4 °C for 2 h, then shaken for 2 min and centrifuged for 20 min at 13 000 rpm. The supernatant (500 µL) was derivatized by the addition of 300 µL distilled water, 300 µL isopropanol, 200 µL pyridine and 100 µL propyl chloroformat. Then, the samples were shaken vigorously for 2 min. Hexane (300 µL) was added to the reaction mixture and vortexed for 1 min followed by centrifugation at 13 000 rpm for 5 min. An aliquot of 300 µL derivative extraction (upper hexane layer) was transferred to a sample vial. The extraction procedure was then repeated by adding 200 µL hexane to the reaction mixture. Another 200 µL aliquot of derivative extraction was transferred to the sample vial with the first extraction for analysis. For the culture supernatant quantitation, 500 µL of culture supernatant was extracted 500 µL of NaOH solution (5 mM), and 10 µL of internal standard (acetic acid‐d₄, 5 mg mL^−1^) followed by homogenize for 5 min. The homogenate was stored at 4 °C for 2 h, then shaken for 2 min and centrifuged for 20 min at 13 000 rpm. Subsequently, supernatant (500 µL) was derivatized using propyl chloroformat. For liver tissue quantitation, 50 mg liver was homogenized with 1 mL NaOH solution (5 mM), and add 10 µL of internal standard (acetic acid‐d₄, 1 mg mL^−1^). The mixture was homogenized for 5 min, stored at 4 °C for 2 h, shaken for 2 min, and then centrifuged at 13 000 rpm for 20 min. 500 µL supernatant was derivatized using propyl chloroformat.

### Molecular Docking

The structure of the HDAC9 catalytic domain was modeled according to the method reported before.^[^ ^60^ ^]^ Briefly, the required sequence (628‐1010) was obtained from the UniPROT database (https://www.uniprot.org). And, its homologous proteins were searched by the means of BLAST algorithm.^[^ ^61^ ^]^ The crystal structure used as the template was retrieved from a protein data bank (www.rcsb.org). Homology models of HDAC9 then were constructed in the homology modeling module of the Schrödinger 2009 platform (Schrödinger, LLC, New York, NY) with default settings, based on a HDAC7 crystal (PDB code: 3C0Y, identity 69.01%), among which a model with best performance in Ramachandran plot assessment was selected. The model structure was optimized using steepest descent and Polak‐Ribiere conjugate gradient algorithms sequentially until a convergence in gradient (0.01) was achieved, which was performed in the MacroModel module of Schrodinger with default settings. The HDAC9 model was prepared with the Protein Preparation Wizard workflow in Schrödinger. And, acetic acid was docked into the Zn^2+^ site using the Glide module in an SP precision with default parameters. The docked pose with the top‐ranked Glide score was selected for further analysis.

### Statistical Analysis

Data are shown as means ± SEM. Multigroup comparisons were performed using one‐way ANOVA (Dunnett's post‐test) or the Kruskal‐Wallis test. Student's t‐test or Mann‐Whitney U test was used for comparisons between 2 groups. At least 3 independent experiments were performed. Analyses were performed with GraphPad Prism Software. Differences with *p* < 0.05 were considered to be statistically significant (^∗^ *p* < 0.05; ^∗^^∗^ *p* < 0.01; ^∗^^∗^^∗^ *p* < 0.001; ns, no significance).

## Conflict of Interest

The authors declare no conflict of interest.

## Author Contributions

Y. Z. and D.X. contributed equally to this work. Y. Z. and W. Z. wrote the manuscript. Y. Z., D. X., X. C., and B. L. performed experiments and interpreted the data. X. X., M. W., and Y. F. conducted animal and mass spectrometry experiments. X. S., A. Y., and Y. Z. recruited subjects and collected samples. H. Y. supervised the project. W. Z. supervised and designed the project. P. L. supervised, designed, and funded the project.

## Supporting information



Supporting Information

## Data Availability

The data are available from the corresponding author on reasonable request.
