# Supplementary material for: Gut Commensal Barnesiella Intestinihominis Ameliorates Hyperglycemia and Liver Metabolic Disorders
Source: Adv Sci (Weinh). 2024 Dec 31;12(8):2411181. doi: 10.1002/advs.202411181 (PMC11848638; doi:10.1002/advs.202411181)
Supplement: Supplementary file 1 — Supporting Information [file ADVS-12-2411181-s001.docx]

**Supporting Information**

**Gut Commensal *Barnesiella intestinihominis* Ameliorates Hyperglycemia and Liver Metabolic Disorders**

***Ye Zhang****,* ***Dong Xu,*** ***Xuyi Cai, Xue Xing, Xin Shao, Ailing Yin, Yanyan Zhao, Mengyuan Wang, Yu-nuo Fan,*** ***Boao Liu, Hua Yang^*^, Wei Zhou^*^ and Ping Li^*^***

Supplementary Figures


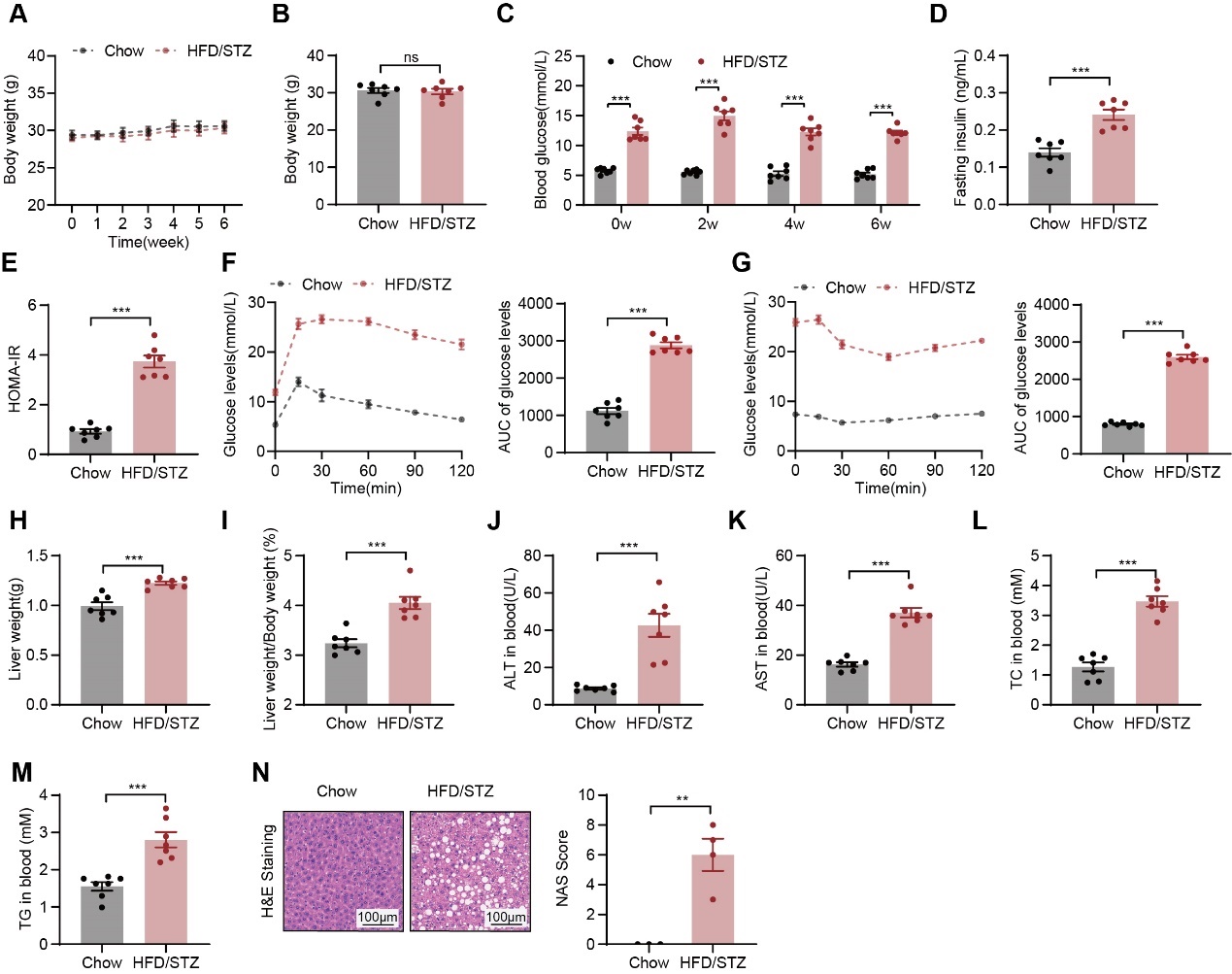


**Figure S1. HFD/STZ-induced T2D mice exhibited hyperglycemia and liver metabolic** disorder. A) Body weight curve (n = 7). B) Body weight at week 6 (n = 7). C) Fasting blood glucose levels at 2, 4 and 6 weeks (n = 7). D) Insulin levels (n = 7). E) HOMA-IR index (n = 7). F-G) OGTT (F) and ITT (G) with AUC (n = 7). H) Liver weight (n = 7). I) **Liver weight/body weight ratio** (n = 7). J-K) ALT and AST levels in blood (n = 7). L-M) Serum TC and TG levels (n = 7). N) Representative photomicrographs of liver H&E staining and histological scores (scale bar, 100 μm, n = 3-4). Data were shown as mean ± SEM. Statistical analysis was performed by two-tailed Student’s t-test (B-N). ***p*<0.01; ***: *p*<0.001, ns: no significance.


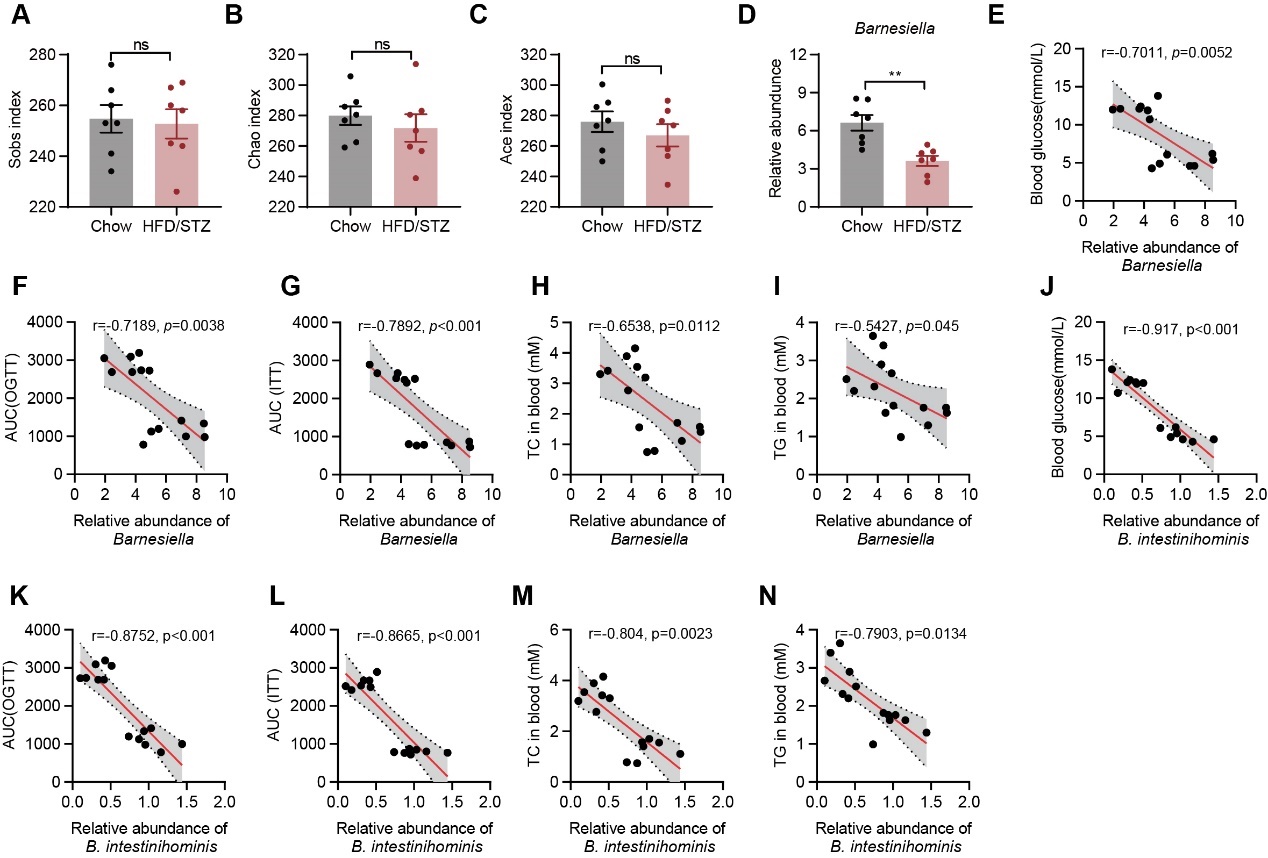


**Figure S2.** The abundance of *B. intestinihominis* was associated with the pathological progression of T2D mice. A-C) α-diversity indicated by the Sobs, Chao, and ACE index (n = 7). D) The abundance of *Barnesiella* assessed by 16S rRNA **gene** sequencing (n = 7). E-I) Pearson’s analysis of the correlations between the fecal ***Barnesiella*** abundance and metabolic parameters. J-N) Pearson’s analysis of the correlations between the fecal *B. intestinihominis* abundance and metabolic parameters. Data were shown as mean ± SEM. Statistical analysis was performed by two-tailed Student’s t-test (A-D). ***p*<0.01; ns, no significance.


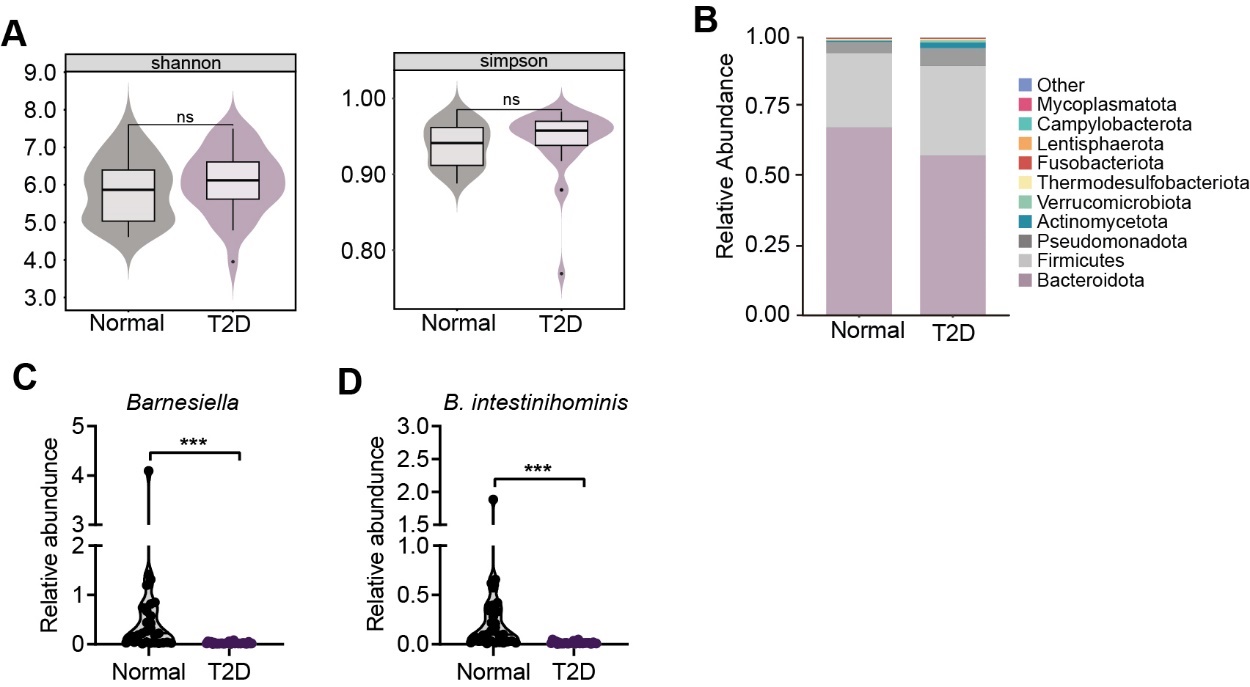


**Figure S3.** The abundance of *B. intestinihominis* was decreased in T2D patients by shotgun metagenomics. A) α-diversity indicated by the Shannon and Simpson index. B) The abundance of bacteria at the phylum level from shotgun metagenomics. C-D) The abundance of *Barnesiella* (C) and *B. intestinihominis* (D) assessed by shotgun metagenomics. Statistical analysis was performed by Mann-Whitney U test. ****p*<0.001; ns, no significance.


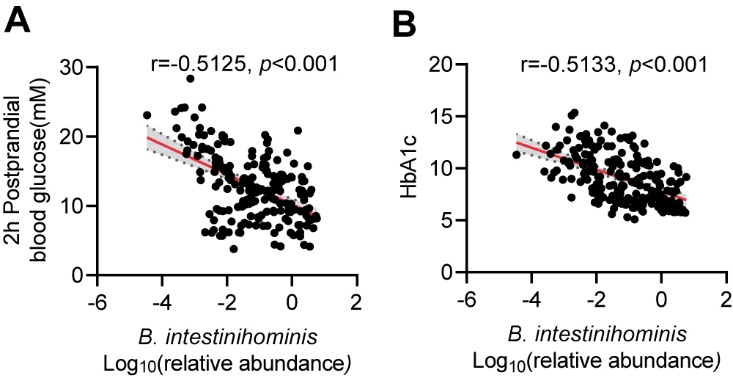


**Figure S4.** Pearson’s analysis of the correlations between the abundance of *B. intestinihominis* and 2hPBG or HbA1c. A) Pearson’s analysis of the correlations between the abundance of *B. intestinihominis* from microbiota discovery set 1-2 and 2hPBG. B) Pearson’s analysis of the correlations between the abundance of *B. intestinihominis* from microbiota discovery set 1-2 and HbA1c.


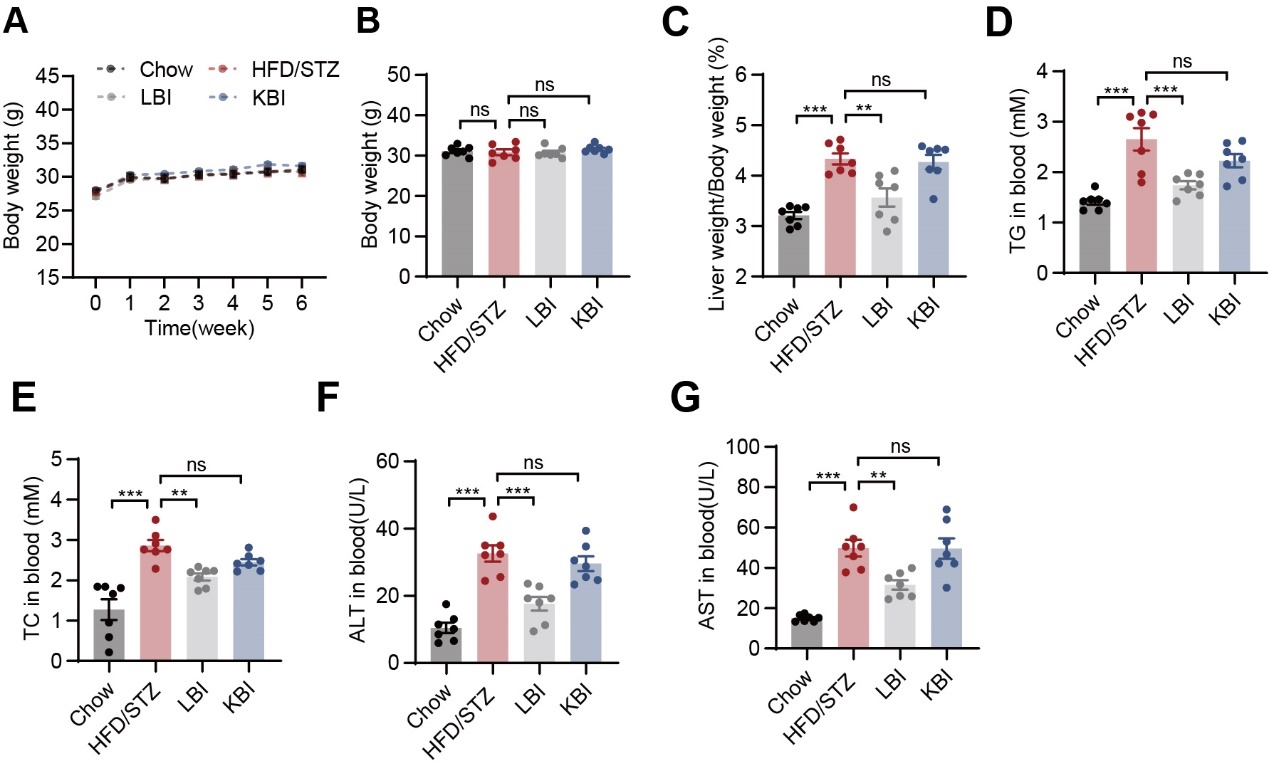


**Figure S5.** Oral administration of LBI attenuates metabolic disorder in HFD/STZ mice. A) Body weight curve (n = 7). B) Body weight at week 6 (n = 7). C) **Liver weight/body weight ratio** (n = 7). D-G) TG, TC, ALT, and AST levels in blood (n = 7). Data were shown as mean ± SEM. Statistical analysis was performed by one-way ANOVA with Dunnett’s post-test (B-G). ***p*<0.01; ****p*<0.001; ns, no significance.


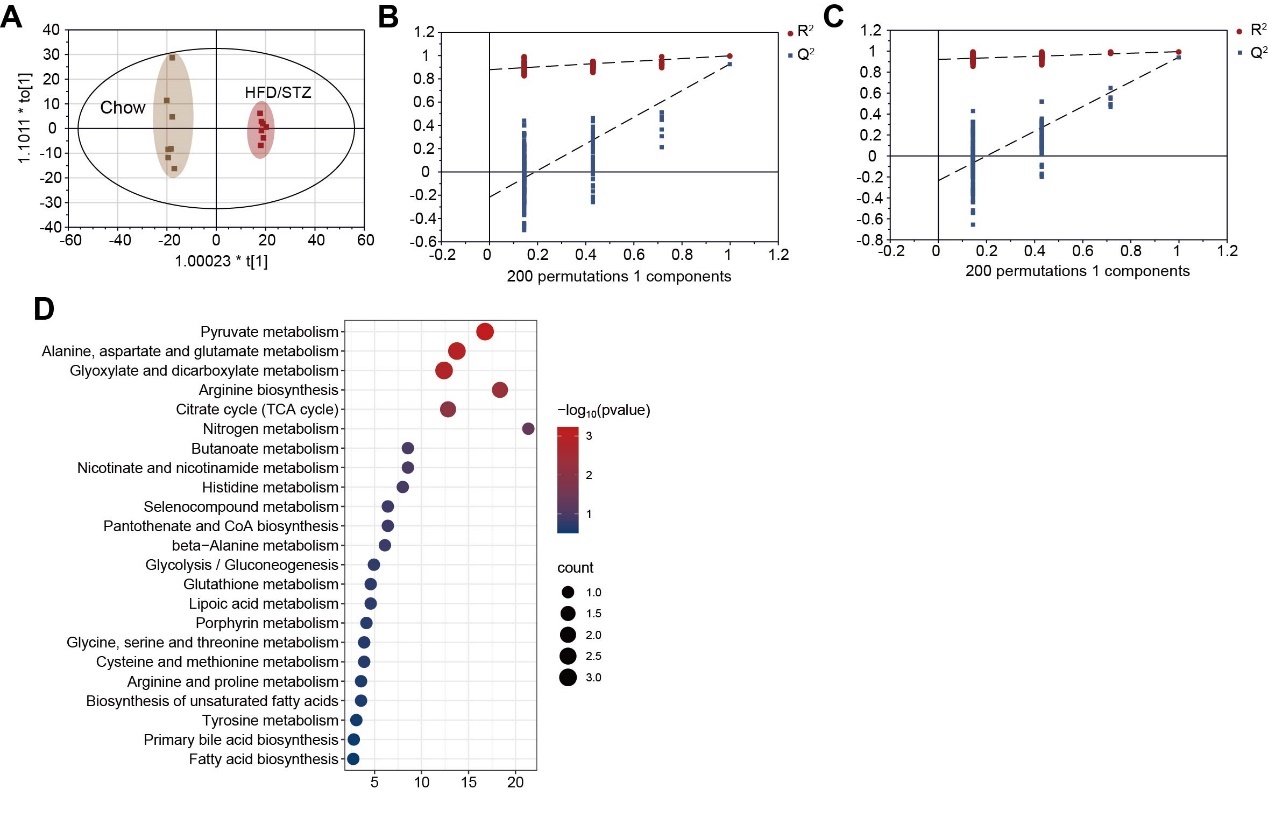


**Figure S6.** Metabolomic analysis identified *B. intestinihominis*-derived metabolites. A) OPLS-DA analysis of metabolic profile in Chow and HFD/STZ groups (n = 7). B-C) 200 OPLS-DA model validations. D) Metabolic pathways in the Chow versus HFD/STZ groups.


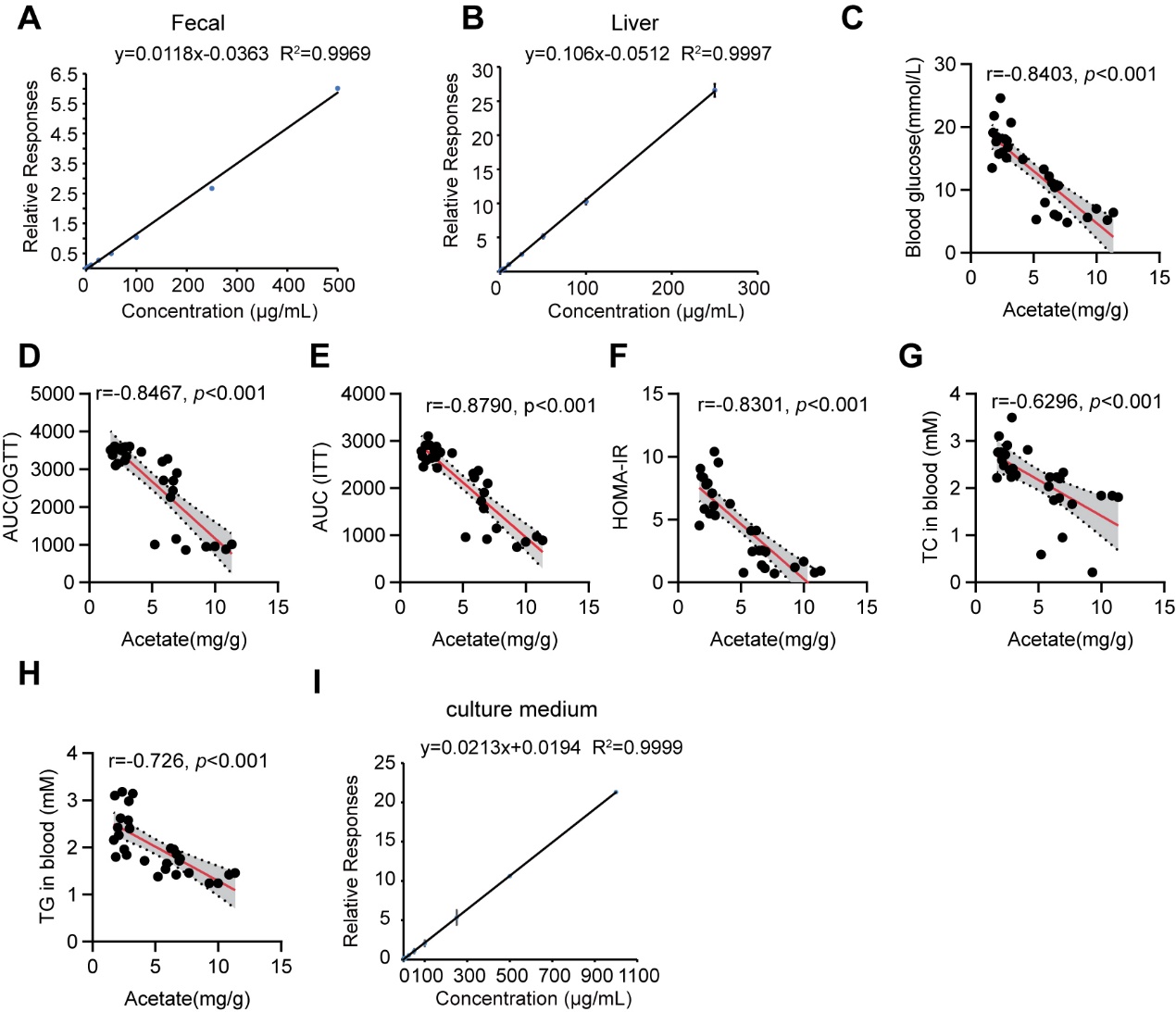


**Figure S7.** Quantitative analysis of acetate was conducted using GC-MS. A-B) Standard curve for quantification of fecal and liver acetate (n = 3). C-H) Pearson’s analysis of the correlations between the fecal acetate levels and metabolic parameters. I) Standard curve for quantification of culture medium supernatant acetate (n = 3).


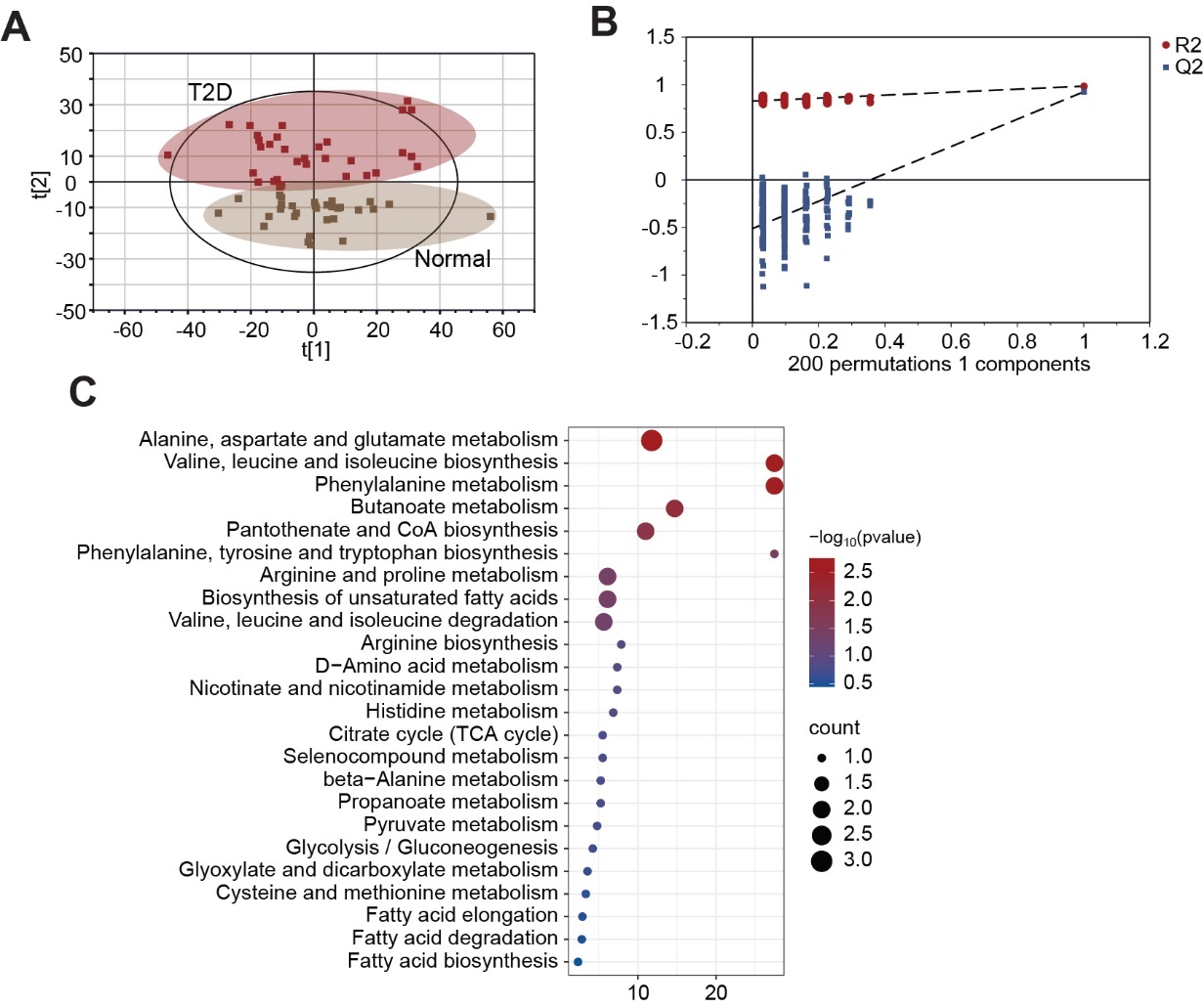


**Figure S8.** Metabolomic analysis of healthy subjects and T2D patients. A) PCA score plots for discriminating the fecal metabolome from healthy subjects (n = 30) and T2D patients (n = 32). B) 200 OPLS-DA model validations. C) Metabolic pathways in the healthy subjects versus T2D patients.


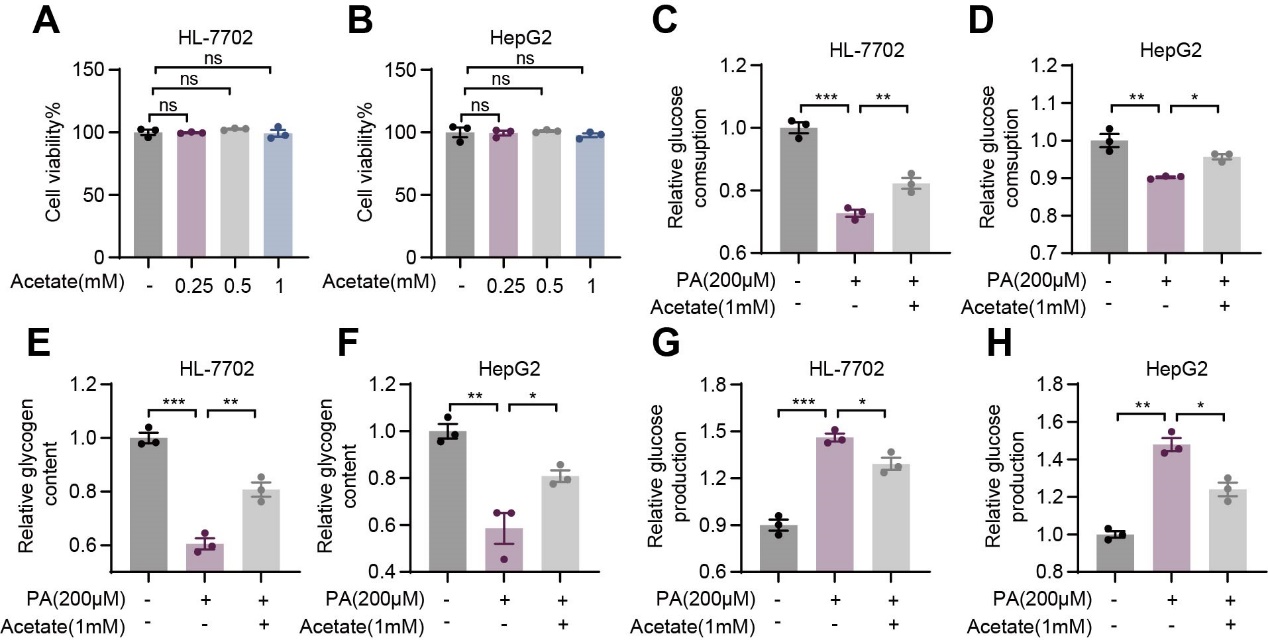


**Figure S9.** Acetate attenuated glucose homeostasis in HepG2 and HL-7702 cells. A-B) Effect of acetate on the viability of HepG2 and HL-7702 cells (n = 3). C-D) The consumption was detected in HepG2 and HL-7702 cells (n = 3). E-F) The glycogen content was detected in HepG2 and HL-7702 cells (n = 3). G-H) The gluconeogenesis was detected in HepG2 and HL-7702 cells (n = 3). Data were shown as mean ± SEM. Statistical analysis was performed by one-way ANOVA with Dunnett’s post-test (A-H). **p*<0.05; ***p*<0.01; ****p*<0.001; ns, no significance.


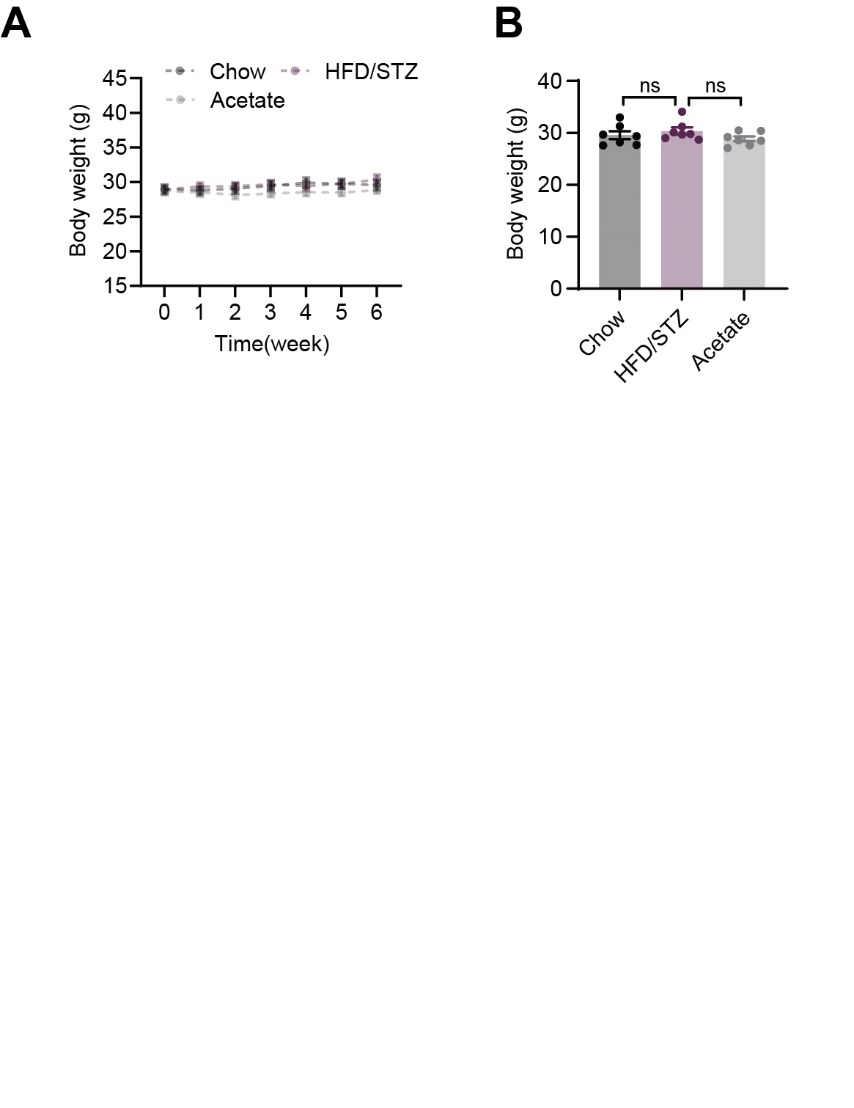


**Figure S10.** The effect of acetate on the body weight of HFD/STZ mice. A) Body weight curve (n = 7). B) Body weight at week 6 (n = 7). Data were shown as mean ± SEM. Statistical analysis was performed by one-way ANOVA with Dunnett’s post-test (B). ns, no significance.


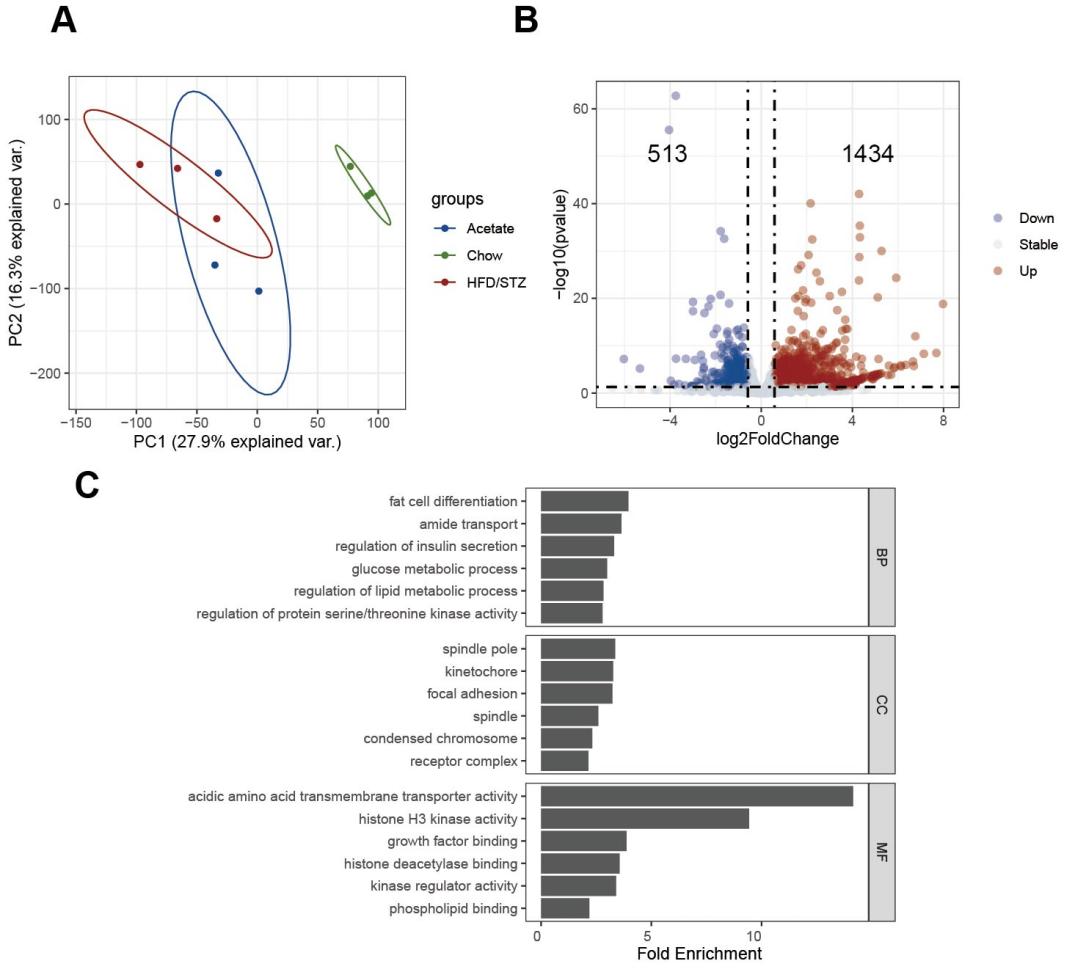


**Figure S11.** Transcriptomic sequencing of liver after acetate administration. A) PCA score plots for discriminating the liver gene from Chow, HFD/STZ, and acetate groups (n = 3). B) Volcano plot shows the number of differential gene downregulated or upregulated by HFD/STZ compared with Chow groups (n = 3). C) Gene ontology analysis in the Chow versus HFD/STZ groups.


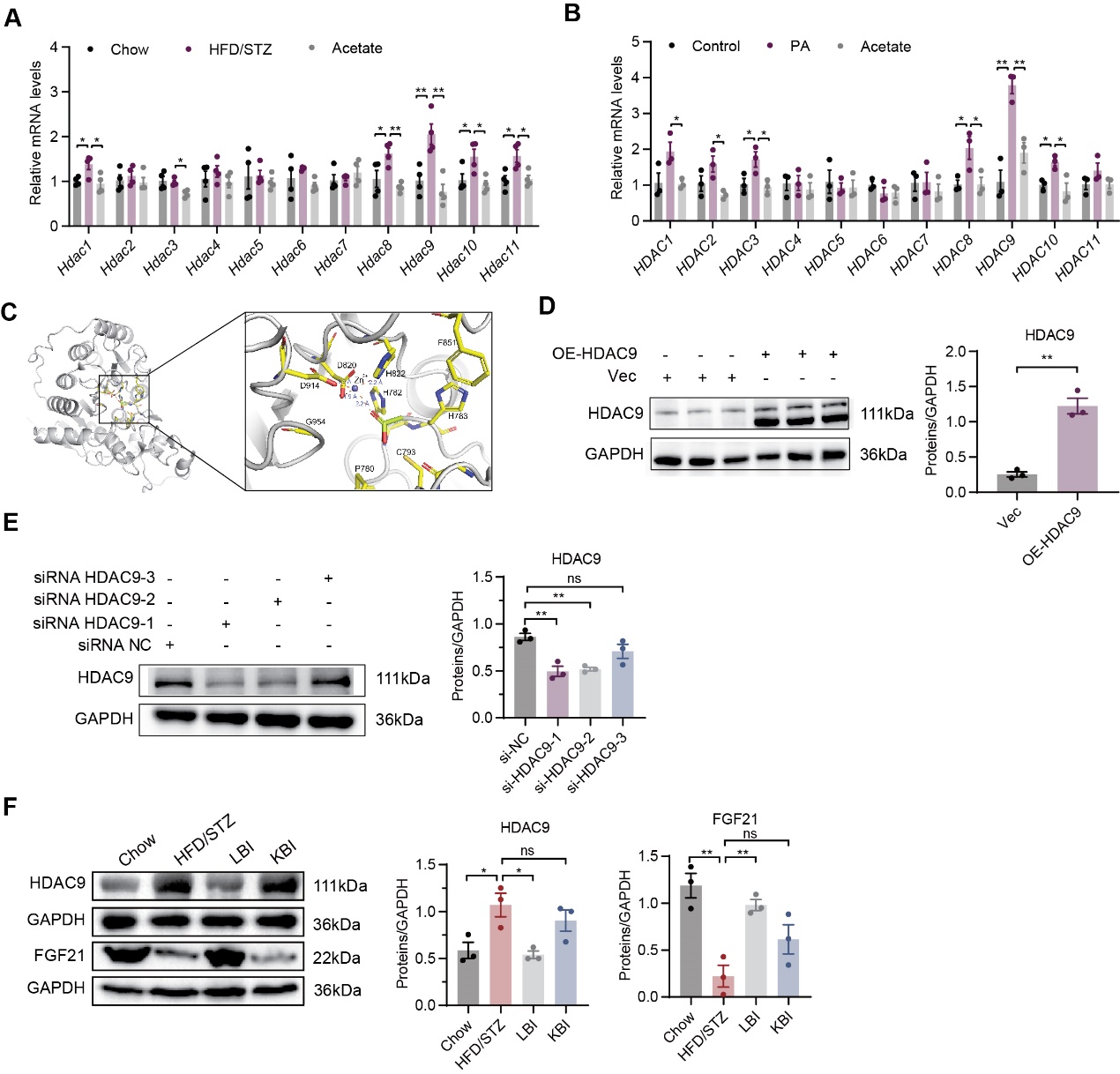
**Figure S12.** Acetate activated FGF21 gene transcription through HDAC9. A) The mRNA expression of *Hdac1-11* in liver (n = 4). B) The mRNA expression of *HDAC1-11* in HepG2 cells (n = 3). C) Binding mode of acetate (limon stick) in HDAC9 (limon stick and grey cartoon) from the molecular docking result. Acetate fits in the zinc ion site and approaches the zinc ion with a distance of 2.2 Å, equivalent to the length of chelate bond between zinc ion and H822. D) Validation of the efficiency of overexpression of HDAC9 (n = 3). E) Effect of different siRNAs on expression of HDAC9 in HepG2 cells (n = 3). F) Liver expression of HDAC9 and FGF21 after LBI and KBI administration (n = 3). Data were shown as mean ± SEM. Statistical analysis was performed by one-way ANOVA with Dunnett’s post-test (A-B and E-F) or two-tailed Student’s t-test (D). **p*<0.05; ***p*<0.01; ns, no significance.


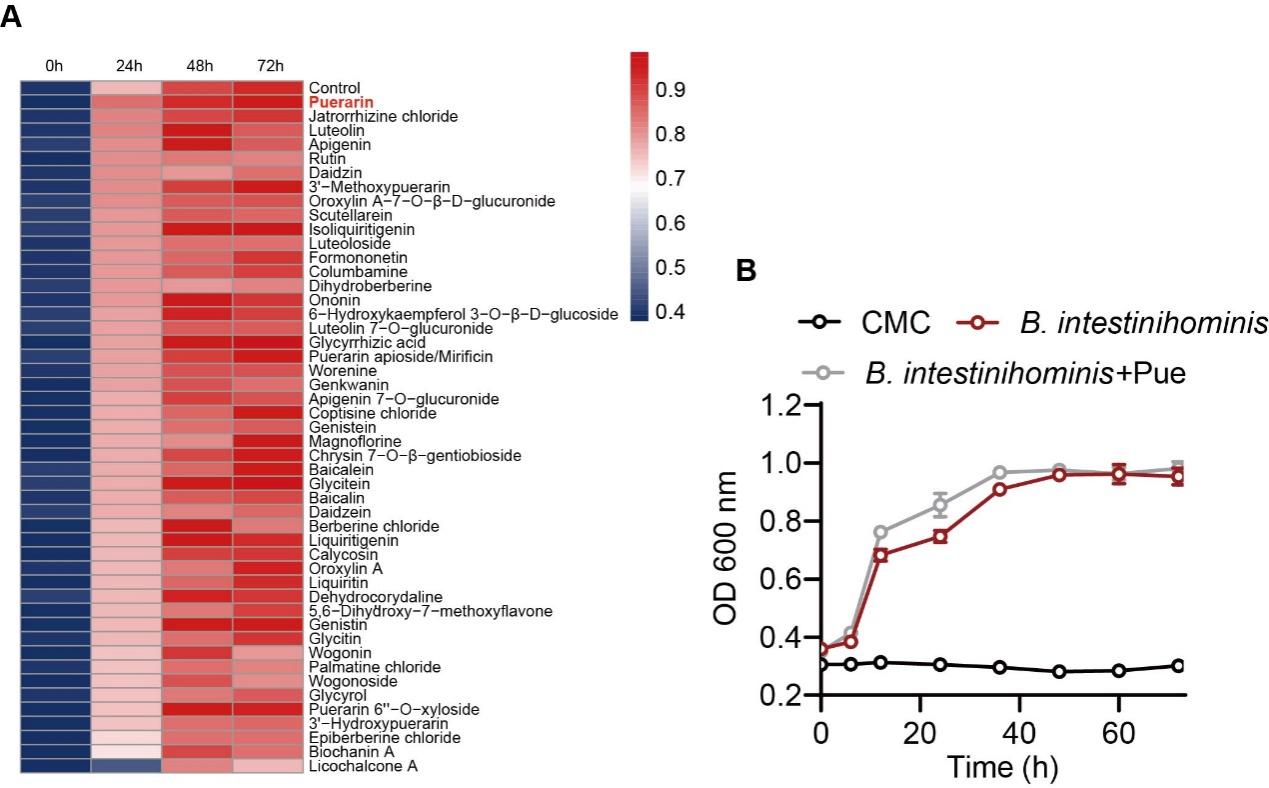


**Figure S13.** *In vitro* screening of prebiotics from 48 active components that promoted the growth of *B. intestinihominis.* A) Heatmap showing the effects of 48 active components on the growth of *B. intestinihominis in vitro* (n = 3)*.* B) The growth curve of *B. intestinihominis* induced by puerarin at indicated time points (n = 3). Data were shown as mean ± SEM.


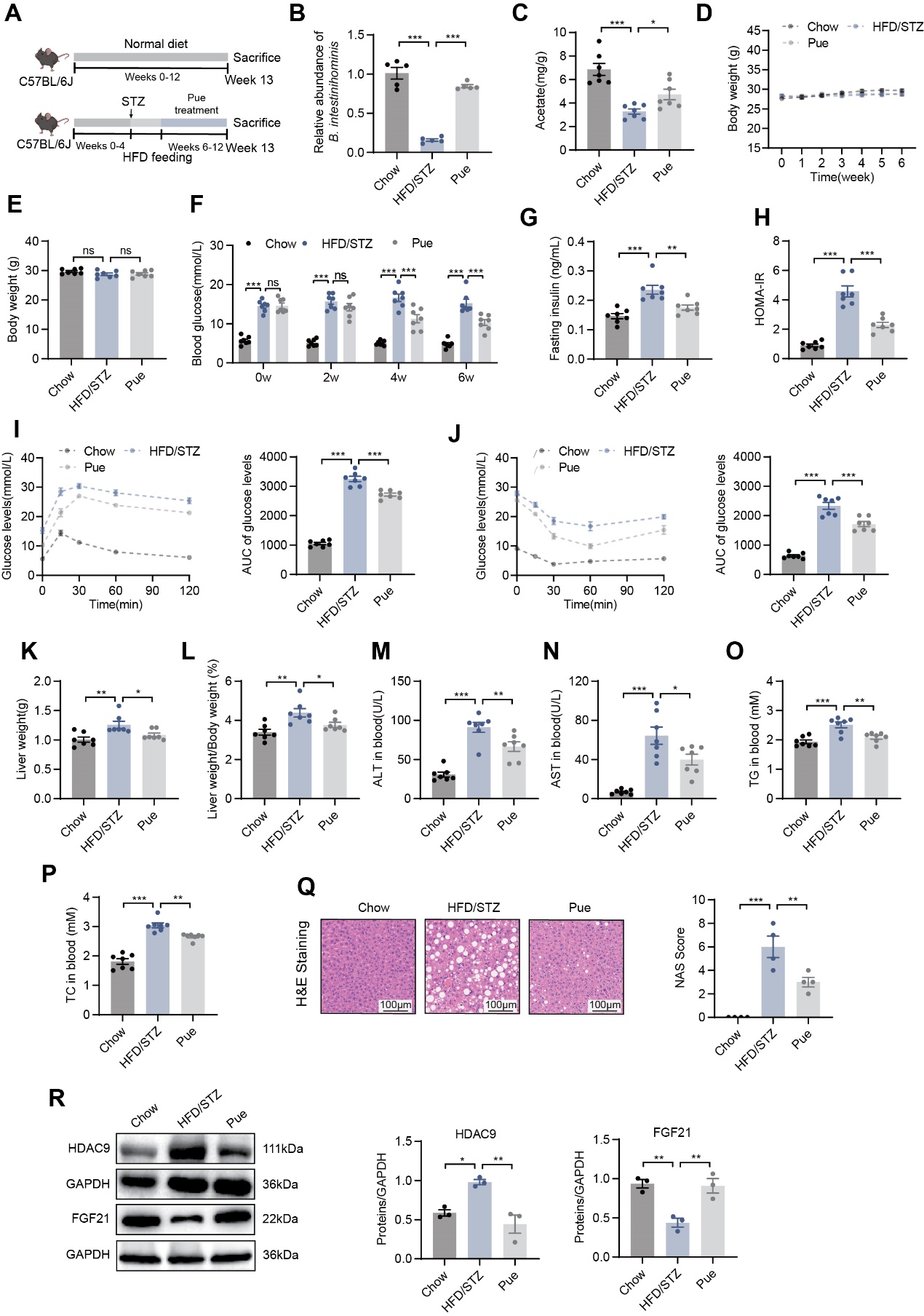


**Figure S14.** Puerarin attenuated hyperglycemia and liver metabolic disorders in HFD/STZ mice. A) A schematic diagram showing the procedure of HFD/STZ mice treated with puerarin. B) The abundance of *B. intestinihominis* assessed by qPCR (n = 5). C) Acetate levels in fecal (n = 7). D) Body weight curve (n = 7). E) Body weight at week 6 (n = 7). F) Fasting blood glucose levels at 2, 4 and 6 weeks (n = 7). G) Insulin levels (n = 7). H) HOMA-IR index (n = 7). I-J) OGTT (I) and ITT (J) with AUC (n = 7). K) Liver weight (n = 7). L) **Liver weight/body weight ratio** (n = 7). M-P) ALT(M), AST(N), TG(O), and TC(P) levels in blood (n = 7). Q) Representative photomicrographs of liver H&E staining and histological scores (scale bar, 100 μm, n=4). R) Liver expression of HDAC9 and FGF21 assayed by western blot and quantitation using Image J software (n = 3). Data were shown as mean ± SEM. Statistical analysis was performed by one-way ANOVA with Dunnett’s post-test (B, C and E-R). **p*<0.05; ***p*<0.01; ****p*<0.001; ns, no significance.


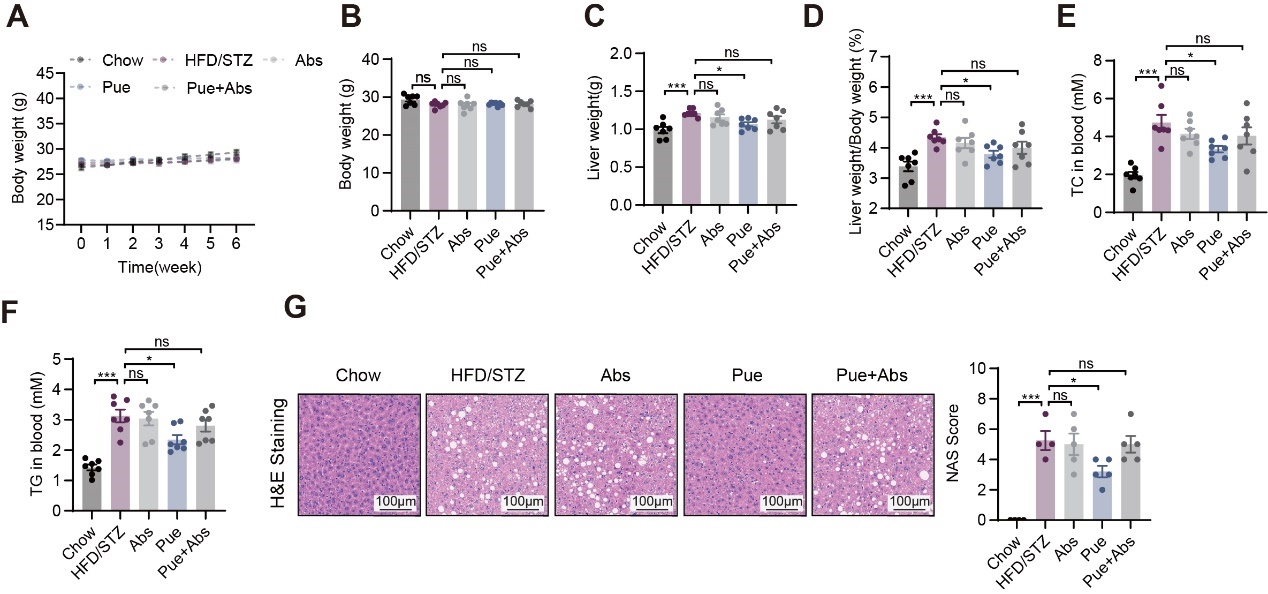


**Figure S15.** Puerarin attenuated metabolic disorders in a gut microbiota–dependent manner. A) Body weight curve (n = 7). B) Body weight at week 6 (n = 7). C) Liver weight (n = 7). D) **Liver weight/body weight ratio** (n = 7). E-F) TC(E) and TG(F) levels in blood (n = 7). G) Representative photomicrographs of liver H&E staining and histological scores (scale bar, 100 μm, n=4-5). Data were shown as mean ± SEM. Statistical analysis was performed by one-way ANOVA with Dunnett’s post-test (B-G). **p*<0.05; ****p*<0.001; ns, no significance.


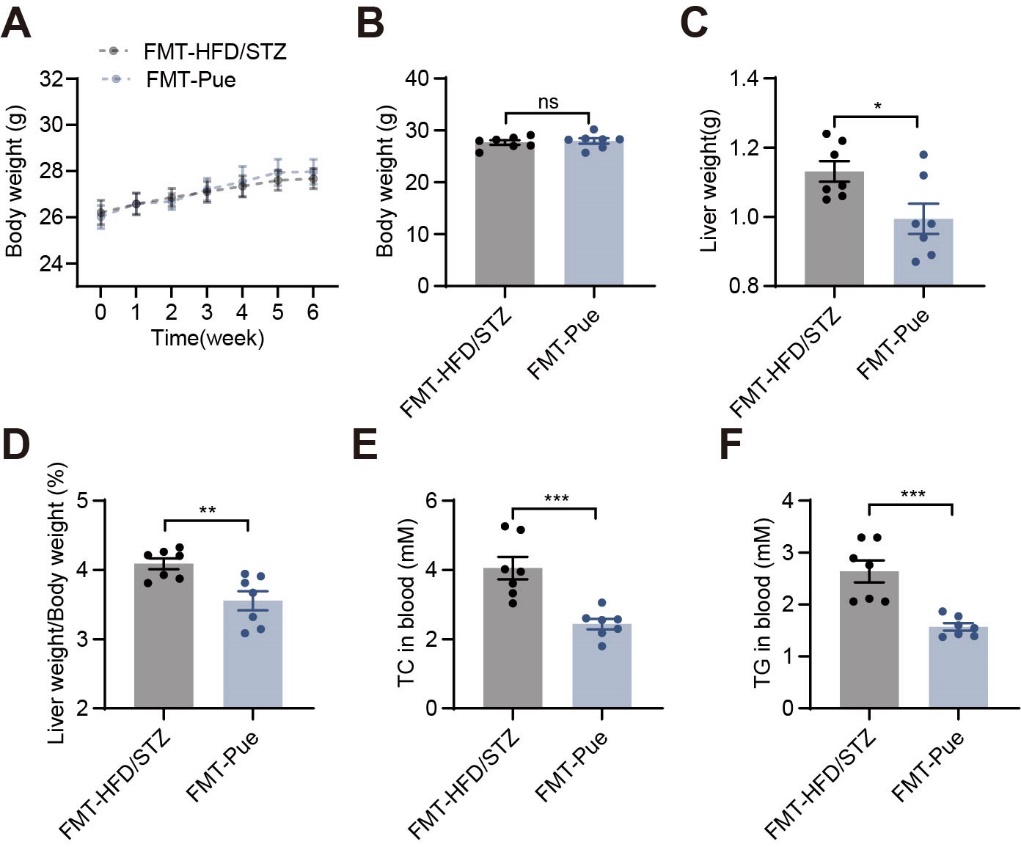


**Figure S16.** Fecal transplants of puerarin exhibited metabolic protection in HFD/STZ mice. A) Body weight curve (n = 7). B) Body weight at week 6 (n = 7). C-D) Liver weight and **liver weight/body weight ratio** (n = 7). E-F) TC(E) and TG(F) levels in blood (n = 7). Data were shown as mean ± SEM. Statistical analysis was performed by two-tailed Student’s t-test (B-F). **p*<0.05, ***p*<0.01; ****p*<0.001; ns, no significance.


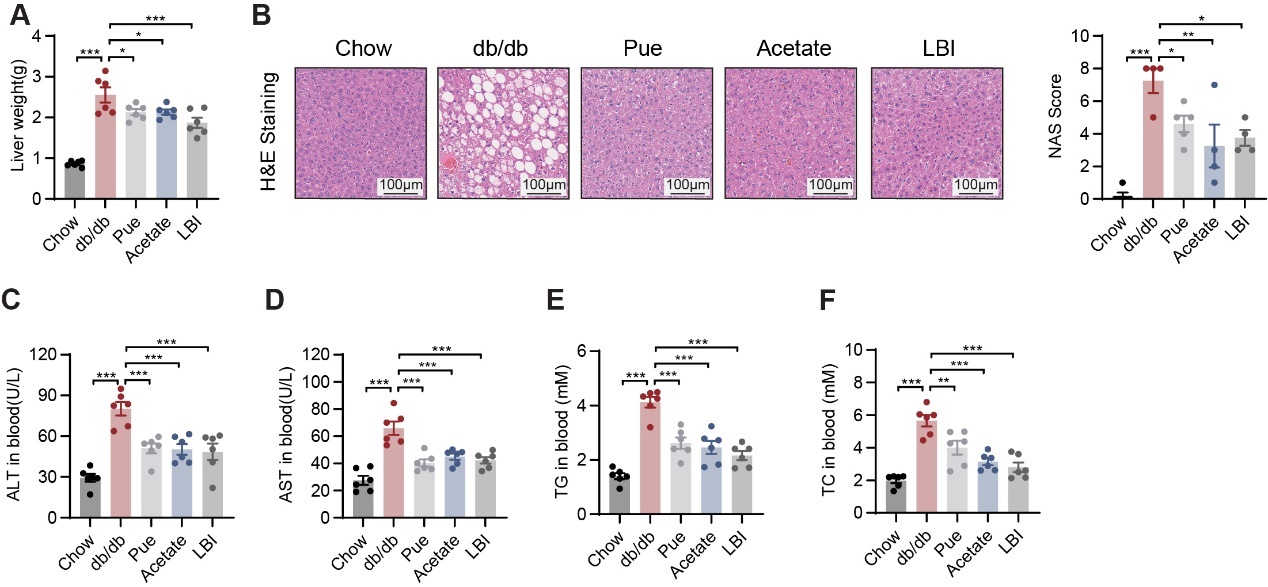


**Figure S17.** LBI, acetate and puerarin attenuated **liver metabolic disorders** in db/db mice. A) Liver weight (n = 6). B) Representative photomicrographs of liver H&E staining and histological scores (scale bar, 100 μm, n=4-5). C-F) ALT, AST, TG, and TC levels in blood (n = 6). Data were shown as mean ± SEM. Statistical analysis was performed by one-way ANOVA with Dunnett’s post-test. **p*<0.05; ***p*<0.01; ****p*<0.001.

Supplementary Table

**Table S1. Baseline characteristics of T2D patients and healthy subjects from Nanjing Hospital of Chinese Medicine affiliated to Nanjing University of Chinese Medicine (microbiota discovery set).**

| Characteristics | Healthy subjects(*n*=30) | T2DM(*n*=31) |
| --- | --- | --- |
| Female, n (%) | 15 (50) | 11 (35) |
| Age (years) | 57±4 | 61±3 |
| BMI (Kg/m^2^) | 21.30±0.52 | 24.76±0.56 |
| 2hPBG (mmol/L) | N/A | 13.82±0.86 |
| HbA1c (%) | N/A | 9.18±0.41 |
| TC (mmol/L) | N/A | 4.572±0.23 |
| TG (mmol/L) | N/A | 1.58±0.18 |
| HDL-C (mmol/L) | N/A | 1.24±0.09 |
| LDL-C (mmol/L) | N/A | 2.84±0.20 |

Data were shown as mean ± SEM. BMI: body mass index; 2hPBG: 2 hours postprandial blood glucose; HbA1c: Hemoglobin A1c; TC: total cholesterol; TG: triglyceride; HDL-C: high-density lipoprotein cholesterol; LDL-C: low-density lipoprotein cholesterol.

**Table S2. Baseline characteristics of T2D patients and healthy subjects (microbiota validation sets).**

| Characteristics | Healthy subjects  (*n*=103) | T2DM (*n*=150, microbiota  validation set1) | T2DM (*n*=57, microbiota  validation set 2) |
| --- | --- | --- | --- |
| Female, n (%) | 43 (42) | 50 (33) | 17 (30) |
| Age (years) | 51±1 | 58±2 | 51±2 |
| BMI (Kg/m^2^) | 21.73±0.25 | 25.12±0.44 | 25.42±0.56 |
| 2hPBG (mmol/L) | N/A | 11.42±0.35 | 16.33±0.53 |
| HbA1c (%) | N/A | 9.22±0.30 | 9.04±0.30 |
| TC (mmol/L) | N/A | 4.93±0.13 | 4.50±0.14 |
| TG (mmol/L) | N/A | 1.94±0.13 | 1.96±0.23 |
| HDL-C (mmol/L) | N/A | 1.17±0.03 | 1.03±0.04 |
| LDL-C (mmol/L) | N/A | 2.56±0.14 | 2.70±0.13 |

Data were shown as mean ± SEM. BMI: body mass index; 2hPBG: 2 hours postprandial blood glucose; HbA1c: Hemoglobin A1c; TC: total cholesterol; TG: triglyceride; HDL-C: high-density lipoprotein cholesterol; LDL-C: low-density lipoprotein cholesterol.

**Table S3. Differential metabolites in Chow *vs* HFD/STZ group.**

| **Rt(min)** | ***m/z*** | **Metabolites** | **HMDB ID** | ***p*** | **FDR-*p*** | **VIP** |
| --- | --- | --- | --- | --- | --- | --- |
| 3.02^*^ | 43.0 | Acetate | HMDB0000042 | 1.96E-06 | 4.46E-05 | 1.54 |
| 5.21^*^ | 88.9 | Isobutyrate | HMDB0001873 | 4.57E-03 | 1.58E-02 | 1.21 |
| 5.51 | 43.1 | Carbonic acid | HMDB0000595 | 6.85E-04 | 3.83E-03 | 1.36 |
| 5.88^*^ | 84.9 | Isovaleric acid | HMDB0000718 | 1.82E-02 | 4.81E-02 | 1.08 |
| 5.95^*^ | 71.0 | Butyric acid | HMDB0000039 | 2.79E-05 | 3.23E-04 | 1.47 |
| 6.73 | 84.9 | Valerate | HMDB0000892 | 4.25E-03 | 1.53E-02 | 1.19 |
| 10.37 | 114.8 | Glutarate | HMDB0000661 | 1.59E-03 | 7.07E-03 | 1.33 |
| 12.42 | 140.8 | Fumarate | HMDB0000134 | 2.14E-04 | 1.52E-03 | 1.42 |
| 12.93 | 129.9 | L-Alanine | HMDB0000161 | 3.95E-04 | 2.53E-03 | 1.43 |
| 13.16 | 127.8 | L-Proline | HMDB0000162 | 1.32E-02 | 3.71E-02 | 1.07 |
| 14.56 | 43.1 | Glycine | HMDB0000123 | 7.71E-03 | 2.37E-02 | 1.20 |
| 17.46 | 187.8 | L-Aspartic acid | HMDB0000191 | 4.75E-04 | 2.88E-03 | 1.36 |
| 18.52 | 88.9 | Malic acid | HMDB0000156 | 2.58E-04 | 1.77E-03 | 1.39 |
| 19.03 | 74.0 | Octanoic acid | HMDB0000482 | 1.29E-05 | 1.77E-04 | 1.51 |
| 19.46 | 201.8 | L-Glutamic acid | HMDB0000148 | 4.28E-03 | 1.54E-02 | 1.29 |
| 19.83 | 100.8 | L-Methionine | HMDB0000696 | 2.22E-03 | 9.22E-03 | 1.32 |
| 20.12 | 129.9 | L-NorValine | HMDB0013716 | 5.71E-04 | 3.37E-03 | 1.33 |
| 23.44 | 60.9 | Pentadecanoic acid | HMDB0000826 | 6.30E-04 | 3.63E-03 | 1.36 |
| 26.27 | 73.0 | 2-Heptenoic acid | HMDB0031484 | 9.98E-03 | 2.95E-02 | 1.21 |
| 28.60 | 61.0 | Octadecanoic acid | HMDB0000827 | 2.55E-06 | 5.28E-05 | 1.55 |

*: Represents metabolites identified by the reference compounds.

**Table S4. Differential metabolites in HFD/STZ *vs* LBI group.**

| **Rt(min)** | ***m/z*** | **Metabolites** | **HMDB ID** | ***p*** | **FDR-*p*** | **VIP** |
| --- | --- | --- | --- | --- | --- | --- |
| 3.02^*^ | 43.0 | Acetate | HMDB0000042 | 1.74E-06 | 2.98E-05 | 1.48 |
| 5.21^*^ | 88.9 | Isobutyrate | HMDB0001873 | 3.94E-03 | 1.19E-02 | 1.16 |
| 5.51 | 43.1 | Carbonic acid | HMDB0000595 | 1.04E-03 | 4.12E-03 | 1.27 |
| 6.73 | 84.9 | Valerate | HMDB0000892 | 3.20E-05 | 2.44E-04 | 1.39 |
| 10.37 | 114.8 | Glutarate | HMDB0000661 | 2.35E-05 | 1.98E-04 | 1.43 |
| 12.42 | 140.8 | Fumarate | HMDB0000134 | 3.95E-03 | 1.19E-02 | 1.17 |
| 12.93 | 129.9 | L-Alanine | HMDB0000161 | 4.98E-05 | 3.40E-04 | 1.41 |
| 14.56 | 43.1 | Glycine | HMDB0000123 | 1.28E-03 | 4.84E-03 | 1.22 |
| 17.46 | 187.8 | L-Aspartic acid | HMDB0000191 | 7.64E-04 | 3.16E-03 | 1.28 |
| 18.52 | 88.9 | Malic acid | HMDB0000156 | 8.71E-04 | 3.55E-03 | 1.26 |
| 19.03 | 74.0 | Octanoic acid | HMDB0000482 | 1.58E-05 | 1.50E-04 | 1.49 |
| 19.46 | 201.8 | L-Glutamic acid | HMDB0000148 | 4.11E-04 | 1.90E-03 | 1.35 |
| 19.83 | 100.8 | L-Methionine | HMDB0000696 | 2.06E-05 | 1.83E-04 | 1.45 |
| 20.12 | 129.9 | L-NorValine | HMDB0013716 | 3.34E-04 | 1.63E-03 | 1.32 |
| 23.41 | 106.9 | 4-Hydroxybenzoic acid | HMDB0000500 | 4.80E-06 | 6.59E-05 | 1.46 |
| 23.44 | 60.9 | Pentadecanoic acid | HMDB0000826 | 6.75E-05 | 4.48E-04 | 1.44 |
| 24.43 | 189.9 | L-homophenylalanine | HMDB0250764 | 3.98E-05 | 2.81E-04 | 1.41 |
| 28.60 | 61.0 | Octadecanoic acid | HMDB0000827 | 1.44E-03 | 5.34E-03 | 1.25 |
| 32.67 | 129.8 | L-tryptophan | HMDB0000929 | 1.38E-05 | 1.35E-04 | 1.42 |

*: Represents metabolites identified by the reference compounds.

**Table S5. Differential metabolites in healthy subjects *vs* T2D patients.**

| **Rt(min)** | ***m/z*** | **Metabolites** | **HMDB ID** | ***p*** | **FDR-*p*** | **VIP** |
| --- | --- | --- | --- | --- | --- | --- |
| 4.55 | 45.0 | Lactic acid | HMDB0000190 | 7.39E-07 | 7.29E-06 | 1.76 |
| 7.14^*^ | 43.1 | Acetate | HMDB0000042 | 7.21E-17 | 8.18E-15 | 2.50 |
| 8.02 | 43.1 | Carbamic acid | HMDB0003551 | 6.52E-10 | 1.06E-08 | 2.06 |
| 10.83^*^ | 89.0 | Isobutyrate | HMDB0001873 | 8.95E-04 | 5.30E-03 | 1.24 |
| 12.23^*^ | 71.1 | Butyric acid | HMDB0000039 | 2.46E-14 | 1.30E-12 | 2.36 |
| 15.80 | 103.0 | Valerate | HMDB0000892 | 2.64E-03 | 1.36E-02 | 1.24 |
| 19.70 | 43.1 | Heptanoic acid | HMDB0000666 | 9.89E-05 | 7.30E-04 | 1.44 |
| 20.76 | 69.0 | Succinic acid | HMDB0000254 | 5.05E-04 | 3.28E-03 | 1.31 |
| 21.05 | 89.0 | Phenylacetic acid | HMDB0000209 | 5.14E-03 | 2.35E-02 | 1.33 |
| 21.38 | 71.1 | L-Alanine | HMDB0000161 | 3.86E-05 | 3.07E-04 | 1.55 |
| 22.04 | 206.0 | L-Valine | HMDB0000883 | 1.65E-11 | 4.52E-10 | 2.25 |
| 22.60 | 220.8 | L-Leucine | HMDB0000687 | 6.99E-03 | 3.02E-02 | 1.06 |
| 22.84 | 113.1 | D-proline | HMDB0003411 | 7.60E-03 | 3.25E-02 | 1.12 |
| 23.37 | 80.0 | L-Aspartic acid | HMDB0000191 | 8.97E-13 | 3.31E-11 | 2.26 |
| 23.60 | 60.0 | L-Methionine | HMDB0000696 | 8.66E-05 | 6.49E-04 | 1.50 |
| 23.85 | 61.0 | L-Phenylalanine | HMDB0000159 | 8.38E-03 | 3.50E-02 | 1.06 |
| 24.03 | 68.0 | L-proline | HMDB0000162 | 1.29E-03 | 7.24E-03 | 1.30 |
| 24.27 | 61.0 | Palmitic acid | HMDB0000220 | 1.23E-10 | 2.48E-09 | 2.12 |
| 25.22 | 61.0 | Octadecanoic acid | HMDB0000827 | 2.18E-04 | 1.52E-03 | 1.40 |

*: Represents metabolites identified by the reference compounds.

**Table S6**. **Characteristics of 48 natural compounds**

| **CAS number** | **Name** | **Molecular formula** | **Molecular weight** |
| --- | --- | --- | --- |
| 117047-07-1 | 3'-Methoxypuerarin | C_22_H_22_O_10_ | 446.4 |
| 21967-41-9 | Baicalin | C_21_H_18_O_11_ | 446.36 |
| 529-53-3 | Scutellarein | C_15_H_10_O_6_ | 286.24 |
| 437-64-9 | Genkwanin | C_16_H_12_O_5_ | 284.27 |
| 3621-36-1 | Columbamine | C_20_H_20_NO_4_^+^ | 338.38 |
| 29550-13-8 | 5,6-Dihydroxy-7-methoxyflavone | C_16_H_12_O_5_ | 284.26 |
| 961-29-5 | Isoliquiritigenin | C_15_H_12_O_4_ | 256.26 |
| 103654-50-8 | Puerarin apioside/Mirificin | C_26_H_28_O_13_ | 548.5 |
| 3681-99-0 | Puerarin | C_21_H_20_O_9_ | 416.4 |
| 36948-76-2 | Oroxylin A-7-*O*-β-D-glucuronide | C_22_H_20_O_11_ | 460.39 |
| 520-36-5 | Apigenin | C_15_H_10_O_5_ | 270.24 |
| 2141-09-5 | Magnoflorine | C_20_H_24_NO_4_^+^ | 342.41 |
| 491-70-3 | Luteolin | C_15_H_10_O_6_ | 286.24 |
| 145134-61-8 | 6-Hydroxykaempferol 3-*O*-β-D-glucoside | C_21_H_20_O_12_ | 464.38 |
| 480-11-5 | Oroxylin A | C_16_H_12_O_5_ | 284.27 |
| 6681-15-8 | Jatrorrhizine chloride | C_20_H_19_NO_4_ | 338.37 |
| 114240-18-5 | Puerarin 6''-*O*-xyloside | C_26_H_28_O_13_ | 548.5 |
| 58749-22-7 | Licochalcone A | C_21_H_22_O_4_ | 338.4 |
| 51059-44-0 | Wogonoside | C_22_H_20_O_11_ | 460.39 |
| 29741-09-1 | Apigenin 7-*O*-glucuronide | C_21_H_18_O_11_ | 446.36 |
| 38763-29-0 | Worenine | C_20_H_16_NO_4_^+^ | 334.34 |
| 483-15-8 | Dihydroberberine | C_20_H_19_NO_4_ | 337.38 |
| 529-59-9 | Genistin | C_21_H_20_O_10_ | 432.38 |
| 578-86-9 | Liquiritigenin | C_15_H_12_O_4_ | 256.26 |
| 6020-18-4 | Coptisine chloride | C_19_H_14_ClNO_4_ | 355.77 |
| 551-15-5 | Liquiritin | C_21_H_22_O_9_ | 418.4 |
| 491-80-5 | Biochanin A | C_16_H_12_O_5_ | 284.27 |
| 446-72-0 | Genistein | C_15_H_10_O_5_ | 270.24 |
| 486-66-8 | Daidzein | C_15_H_10_O_4_ | 254.24 |
| 552-66-9 | Daidzin | C_21_H_20_O_9_ | 416.38 |
| 153-18-4 | Rutin | C_27_H_30_O_16_ | 610.52 |
| 88640-89-5 | Chrysin 7-*O*-β-gentiobioside | C_27_H_30_O_14_ | 578.52 |
| 632-85-9 | Wogonin | C_16_H_12_O_5_ | 284.27 |
| 889665-86-5 | Epiberberine chloride | C_20_H_18_ClNO_4_ | 371.82 |
| 117060-54-5 | 3'-Hydroxypuerarin | C_21_H_20_O_10_ | 432.38 |
| 633-65-8 | Berberine chloride | C_20_H_18_ClNO_4_ | 371.82 |
| 486-62-4 | Ononin | C_2_2H_22_O_9_ | 430.41 |
| 10605-02-4 | Palmatine chloride | C_21_H_22_ClNO_4_ | 387.86 |
| 491-67-8 | Baicalein | C_15_H_10_O_5_ | 270.24 |
| 485-72-3 | Formononetin | C_16_H_12_O_4_ | 268.27 |
| 1405-86-3 | Glycyrrhizic acid | C_42_H_62_O_16_ | 822.94 |
| 23013-84-5 | Glycyrol | C_21_H_18_O_6_ | 366.37 |
| 20575-57-9 | Calycosin | C_16_H_12_O_5_ | 284.27 |
| 30045-16-0 | Dehydrocorydaline | C_22_H_24_NO_4_^+^ | 366.44 |
| 40246-10-4 | Glycitin | C_22_H_22_O_10_ | 446.41 |
| 40957-83-3 | Glycitein | C_16_H_12_O_5_ | 284.27 |
| 5373-11-5 | Luteoloside | C_21_H_20_O_11_ | 448.38 |
| 29741-10-4 | Luteolin 7-*O*-glucuronide | C_21_H_18_O_12_ | 462.36 |

**Table S7. Primer pairs for real-time qPCR**

| **Species** | **Forward（5'->3'）** | **Reverse（5'->3'）** |
| --- | --- | --- |
| ***Bacteria*** |  |  |
| *All bacteria* | ACTCCTACGGGAGGCAGCAGT | GTATTACCGCGGCTGCTGGCAC |
| *B.intestinihominis* | GTGAAACAGACGCTGAGGC | TACCGCTTACATTGTATCGC |
| *B.propionica* | TATTCGCGGTTCTGCTCTGG | CCTTCTGCACCAAGACCGAT |
| *B.viscericola* | ACGTATGCAATCTGCCTGTA | AGGAGTTTGGACCGTGTCT |
| *Acka* | GTCATCGTGTAGTDMABGGHGG | GGTGGRTTGTGMARWGGTGCDA |
| ***Mus musculus*** |  |  |
| *Fgf21* | GTCCTCCAGCAGCAGTTCTC | CCTGGGTGTCAAAGCCTCTA |
| *Action* | CCAGTTGGTAACAATGCCATG | GGCTGTATTCCCCTCCATCG |
| *Hdac1* | GCGCAGACTCAGGGCACCAA | CGGTAGAGACCATAGTTGAGCAGCA |
| *Hdac2* | GCGTACAGTCAAGGAGGCGGC | GCAGCAAGTTATGAGTCATCCGGA |
| *Hdac3* | CGCCTGGCATTGACTCATAGCCT | AGCGGCACATGTCATGCTGGG |
| *Hdac4* | ACCACATGCCCAGCACGGTG | TGCTGCAGTTGCTGCTCCCG |
| *Hdac5* | GGCATGTCAGGCCGGGAACC | TGGAGCTGGGCATGGCTCCT |
| *Hdac6* | TGTGGGGCTTCAAGGGCTGGA | GGGAAGCTGTCATCCCAAAGGCA |
| *Hdac7* | TCCTCCAATGCAGCCCGCTG | TCTGCATGGTGTCCCGGGGG |
| *Hdac8* | TGCCAACACGGCTCGTTGCT | CATTGCGGTCTGGCCGGCAG |
| *Hdac9* | AGCAGTCCCTTACTCAGGCGGA | CGGTCACATTCCCAGCAGGGC |
| *Hdac10* | CCTGGCGCAGGCATTGCATG | GGAGCAGCCACCTGCAGCA |
| *Hdac11* | ACCTGCTGGTGGTGCACACG | AGGGGCCTCAGCACCTTCCT |
| ***Homo sapiens*** |  |  |
| *HDAC1* | CTACTACGACGGGGATGTTGG | GAGTCATGCGGATTCGGTGAG |
| *HDAC2* | ATGGCGTACAGTCAAGGAGG | TGCGGATTCTATGAGGCTTCA |
| *HDAC3* | CCTGGCATTGACCCATAGCC | CTCTTGGTGAAGCCTTGCATA |
| *HDAC4* | GGCCCACCGGAATCTGAAC | GAACTCTGGTCAAGGGAACTG |
| *HDAC5* | TCTTGTCGAAGTCAAAGGAGC | GAGGGGAACTCTGGTCCAAAG |
| *HDAC6* | AAGAAGACCTAATCGTGGGACT | GCTGTGAACCAACATCAGCTC |
| *HDAC7* | GGCGGCCCTAGAAAGAACAG | CTTGGGCTTATAGCGCAGCTT |
| *HDAC8* | TCGCTGGTCCCGGTTTATATC | TACTGGCCCGTTTGGGGAT |
| *HDAC9* | AGTAGAGAGGCATCGCAGAGA | GGAGTGTCTTTCGTTGCTGAT |
| *HDAC10* | CAGTTCGACGCCATCTACTTC | CAAGCCCATTTTGCACAGCTC |
| *HDAC11* | ACCCAGACAGGAGGAACCATA | TGATGTCCGCATAGGCACAG |
| *ACTION* | TGACGTGGACATCCGCAAAG | CTGGAAGGTGGACAGCGAGG |
